# Designing single-atom catalysts: bridging metal–support interaction and adsorption energy optimization

**DOI:** 10.1039/d5sc08100a

**Published:** 2026-01-12

**Authors:** Huaizhen Cui, Jiaqi Zhang, Chen Chen

**Affiliations:** a Engineering Research Center of Advanced Rare Earth Materials, Department of Chemistry, Tsinghua University Beijing 100084 China cchen@mail.tsinghua.edu.cn; b Beijing University of Chemical Technology Beijing 100029 China

## Abstract

Single-atom catalysts (SACs) offer exceptional potential for the oxygen evolution reaction (OER), yet their practical application is hindered by an incomplete understanding of structure–activity relationships at the atomic scale. Traditional descriptors fail to fully explain the adsorption behavior of key oxygen intermediates, creating a fundamental gap in catalyst design. This review addresses this limitation by introducing a “structure–adsorption” framework that clarifies how metal–support interactions (MSIs) can be tuned through coordination engineering, such as spin configuration, axial coordination, and atomic distance. Our analysis demonstrates that optimal OER activity arises from a balance between orbital hybridization and electrostatic effects, providing clear design principles for next-generation SACs aimed at sustainable energy conversion.

## Abstract 2

This review systematically summarizes the recent advances in design and synthesis methods of SACs for the OER, further discusses how the metal–support interaction promotes the OER activity, and elucidates their structure–activity correlation.

## Graphical abstract

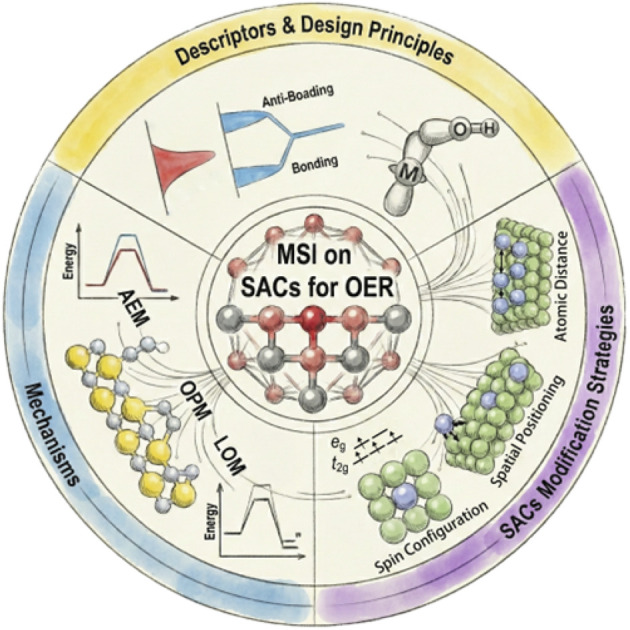

## 1. Introduction

Since Zhang *et al.*^1^ reported an isolated Pt atom catalyst supported on FeO_*x*_ nanocrystals, which exhibited high activity and stability in the CO oxidation reaction, research on SACs has been very active. SACs have broad prospects in both industrial applications and fundamental scientific research. On the one hand, in terms of application value, metal active sites are atomically dispersed and anchored to a support, achieving full utilization of all metal atoms. The single-atom dispersion maximizes the utilization of active sites, enhancing the reactivity while reducing the cost of precious metal catalysts. SACs have been applied in industry, such as a three-way catalyst for vehicle exhaust treatment^2–4^ (Chinese Patent No. CN114682256A, CN114682253A, and CN114939418A), dry reforming of methane and CO_2_, and the synthesis of 1,3-cyclohexanedimethylamine at the ten-thousand-ton scale.^5^ On the other hand, in theoretical research, understanding the relationship between the structure and performance of catalysts at the atomic scale has always been an important goal in catalysis research. SACs provide an opportunity for precise study of structure–activity relationships. In contrast to conventional heterogeneous catalysts with diverse active sites and unclear structure–activity relationships, single-atom catalysts (SACs) present a well-defined coordination structure around isolated metal centers and tunable local environments,^6^ providing an ideal platform for rigorous, in-depth studies at the atomic scale.

Currently, research on SACs is very extensive, with numerous reviews and discussions exploring the precise synthesis,^7,8^ structural characteristics,^9,10^ catalytic performance and mechanisms.^11–13^ However, throughout the entire development process of SACs, whether in the past or now, precisely understanding the structure–activity relationship remains a difficult problem to be solved.

At the single-atom scale, the core of MSI undergoes a fundamental transformation: it is no longer a macroscopic phenomenon related to the physical encapsulation of nanoparticles or interfacial charge transfer, but rather transforms into a direct and strong electronic coupling between a single metal atom and its coordination shell. In SACs, MSI is the core for regulating their microscopic coordination structure. By controlling the microscopic coordination environment, the electronic state and adsorption energy can be altered, thereby changing the catalytic activity. Therefore, MSI has become the primary factor determining the electronic structure, stability, and catalytic performance of SACs.

Electrolytic water splitting for hydrogen production, as a core component of the carbon-neutral energy system, is limited in efficiency by the four-electron transfer kinetic barrier of the OER, with high overpotential leading to significant energy loss.^14,15^ The OER, as a key half-reaction in clean energy technologies such as electrolytic water splitting for hydrogen production and metal–air batteries, has relatively clear and standardized reaction pathways (AEM, LOM, and OPM) and intermediate species (OH*, OOH*, and O*), providing a clear theoretical framework for studying the structure–performance relationship of SACs. Because the reaction pathways and intermediate species of the OER are relatively fixed, external interference factors such as diversity of supports, complex mass transfer, or side reactions are greatly reduced, allowing researchers to focus more on the properties and behaviors of the catalytic sites themselves. This feature makes the OER very suitable for studying the structure–activity relationship of SACs. By precisely regulating the coordination environment, electronic structure of the metal center in SACs and their interaction with the support, the influence on the adsorption energy of specific intermediates can be directly correlated, thereby revealing the intrinsic relationship between the microstructure of SACs and their catalytic performance.^13,16^ At the technical application level, the development of efficient OER catalysts is crucial for enhancing the energy efficiency and economic feasibility of the entire water splitting process. SACs with their extremely high atomic utilization rate and tunable electronic structure have emerged as a promising direction for OER catalyst design. In particular, precious metal-based SACs (such as Ir, Pt, *etc.*) can retain or even enhance the catalytic activity while reducing the amount of precious metals,^17–20^ which hold significant practical application value.

In this review, we propose a “structure–adsorption” theoretical framework to systematically explain the regulation mechanism of MSI in SACs. This framework demonstrates how MSI can enhance OER activity by optimizing the adsorption energy of oxygen intermediates. This is achieved by precisely engineering the single-atom's frontier orbitals—tuning their energy, symmetry, and electron occupancy—through modulation of the local coordination environment. Such orbital engineering governs the degree of hybridization between the single atom and reaction intermediates. Crucially, the adsorption between SACs and oxygen species involves both covalent bonding and electrostatic adsorption. The covalent bond formation is dictated by the three principles of molecular orbital theory: energy alignment, symmetrical matching, and maximum orbital overlap. The electrostatic adsorption arises from the coulombic interaction between the lone pair electrons of the oxygen species and the metal atom. This paper will deeply explore how to finely regulate the orbitals of single-atom active centers through diverse structural engineering strategies such as spin regulation, atomic spacing, and atomic position, thereby optimizing the adsorption energy of key reaction intermediates and ultimately providing a clear roadmap for the development of the next generation of highly efficient and selective SACs.

## 2. The development of metal–support interaction

**Fig. 1:**
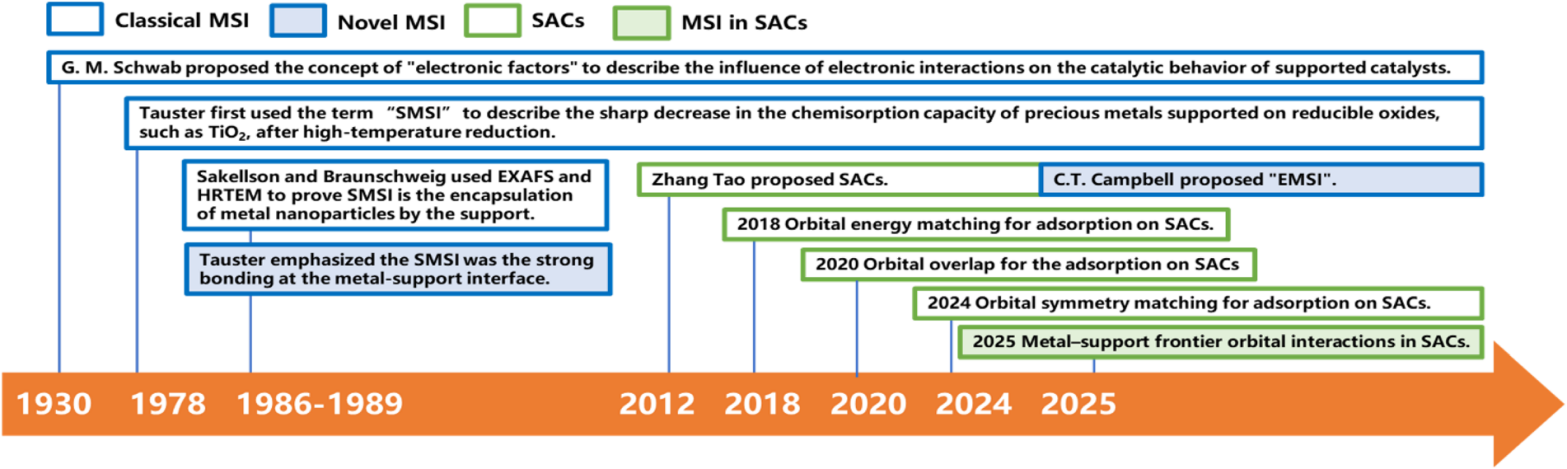
Schematic diagram of the development timeline of MSI.

The research history of MSI is rooted in the exploration of the fundamental principles of heterogeneous catalysis (Fig. 1). Its concept has evolved along with the deepening of theory and the innovation of characterization techniques. As early as the 1930s, G. M. Schwab proposed the concept of “electronic factors”, initially revealing the influence of electronic interactions on the catalytic behavior of supported catalysts, and dividing them into structural effects and synergistic effects.^21^ However, the landmark breakthrough occurred in 1978 when Tauster *et al.*^22^ from ExxonMobil in the United States first used the term “Strong Metal–Support Interaction” (SMSI) to describe a notable phenomenon: after high-temperature reduction treatment (from 200 °C to 500 °C), Group VIII noble metals supported on reducible oxides such as TiO_2_ exhibit a sharp decline in their capacity to chemisorb small molecules like CO and H_2_. Initially, this phenomenon was attributed to electronic perturbations arising from the formation of Pt–Ti chemical bonds. With advances in electron microscopy technology, it was revealed that the essence of SMSI lies in the migration of support species under high-temperature reduction conditions, which encapsulate the metal nanoparticles, forming an overlayer that reduces the adsorption capacity. This encapsulation model gained strong support in 1986 when Sakellson *et al.*,^23^ using extended X-ray absorption fine structure (EXAFS) spectroscopy, provided direct evidence of strong Rh–Ti interfacial bonds in a Rh–TiO_2_ system. It was conclusively established in 1989 following the work of Braunschweig *et al.*,^24^ who employed high-resolution transmission electron microscopy (HRTEM) to directly observe an amorphous support-derived overlayer. It is worth noting that Tauster himself later emphasized that the nature of SMSI should refer to the strong metal–support chemical bond formed at the interface, rather than its subsequent physical and chemical behavior such as encapsulation.^25^ In the 21st century, the concept of MSI has continued to expand. In 2012, Liu *et al.*^26^ first reported the “oxidative strong metal–support interaction” (Oxidative SMSI, O-SMSI) formed between Au and ZnO under oxidative conditions, breaking the traditional perception that SMSI only forms in reducing atmospheres.

Subsequently, in 2017, Tang *et al.*^27^ confirmed that Group IB metals (Au, Ag, Cu, *etc*.) can also have classic SMSI with TiO_2_ under high-temperature reduction conditions. In 2011, Zhang *et al.*^1^ formally proposed the concept of “Single-Atom Catalysts”, and the research on MSI entered a new era at the atomic scale. In the same year, C. T. Campbell proposed the concept of Electronic Metal–Support Interaction (EMSI) to more accurately describe interfacial charge transfer between the metal and the support.^28^ SACs offer an ideal platform for investigating EMSI, as they eliminate complications arising from nanoparticle size and geometric effects.^29^ This model system enables precise investigation of the electronic states of single metal atoms, such as shifts in orbital energy levels induced by interfacial charge transfer. At the atomic scale, engineering the micro-coordination environment of SACs directly tailors the EMSI, which governs catalytic activity and stability through precise charge transfer and modulation of the metal atom's electronic state. These insights provide a fundamental basis for understanding and tailoring catalytic performance through electronic regulation. This development reflects the evolution of MSI research: from initial phenomenological observations to understanding physical origins (encapsulation and interfacial bonding) and ultimately toward precise electronic control (EMSI) at the atomic scale, establishing a coherent multiscale understanding of MSI. Concurrently, the theoretical underpinnings of MSI have been progressively elucidated through extensive research. Orbital hybridization is the fundamental mechanism behind SAC activity, with adsorption free energy representing a macroscopic consequence of these interactions.^30^ By manipulating the micro-coordination environment, it becomes possible to modulate the MSI, thereby tuning the electronic orbital properties of the single metal atom. This in turn affects the interaction between the single atom and adsorbates, leading to tailored catalytic activity. From a quantum chemical perspective, orbital hybridization and frontier orbital interaction theory provide an explanation. Specifically, the frontier orbitals of the metal atom not only hybridize with the orbitals of coordinating atoms from the support but also overlap with the orbitals of reactant or intermediate molecules during catalysis, resulting in the formation of new bonding and antibonding states. The resulting orbital interplay dictates the adsorption configuration and free energy of key species, ultimately controlling the reaction pathway and activation barriers.

## 3. Descriptors for SACs

The performance of SACs is governed not only by the metal center itself but also influenced by the support that surrounds it. Zhang *et al.*^6^ categorized the influence of the support into first shell and outer shell: the first shell directly modulates the electronic structure of the single atom, while the outer shell influences it through electrostatic interactions and steric effects. Thus, MSI is the key to tailor the catalytic properties of SACs. This section will discuss two types of descriptors: one directly quantifying the strength of MSI and the other reflecting how MSI affects the adsorption behavior of the active site.

### 3.1 Descriptors for the strength of MSI

**Fig. 2:**
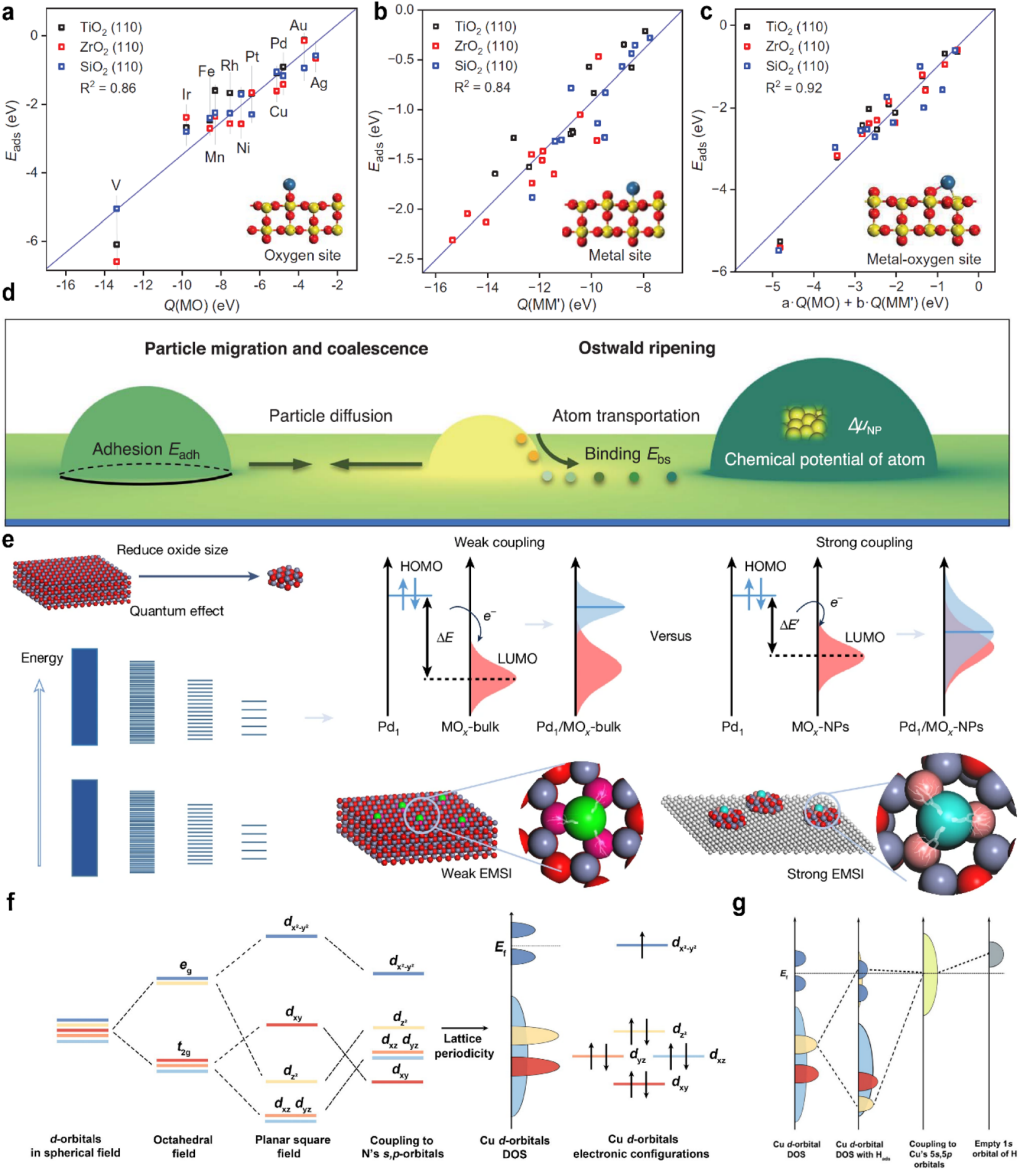
(a) Correlation between the adsorption energy *E*_ads_ of metal atoms bonding only to oxygen atoms of rutile TiO_2_(110), ZrO_2_(110), and SiO_2_(110) surfaces against *Q*(MO), with schematic structures shown in insets. (b) *E*_ads_ of metal atoms bonding only to metal atoms of oxide surfaces against *Q*(MM′). (c) *E*_ads_ of metal atoms bonding to both metal and oxygen atoms on oxide surfaces against *Q*(MO) and *Q*(MM′). Reproduced with permission.^31^ Copyright 2024, The American Association for the Advancement of Science. (d) Schematic of PMC and OR of supported metal NPs. Reproduced with permission.^33^ Copyright 2021, The American Association for the Advancement of Science. (e) Schematic of tailoring EMSIs in Pd_1_/MO_*x*_ SACs by changing the energy band structures of oxide supports by size variation. Reproduced with permission.^35^ Copyright 2025, Springer Nature. (f) Schematic diagram showing the evolution of the d-orbital energy level from degeneracy to that in M–N–C SACs' configuration. And the specific situation in Cu–N–C is given with the consideration of Cu electronic configurations. (g) Schematic representation illustrating the Cu d orbitals' energy level evolution in H–Cu–N–C from the interaction between d orbitals in Cu–N–C and the vacant s orbital of the proton. Reproduced with permission.^36^ Copyright 2025, American Chemical Society.

MSI refers to the physicochemical interplay between the supported metal species and the substrate, the strength of which directly governs the stability of single atoms. Only by forming strong bonds with the support can the metal atom avoid aggregation. In a 2024 study, Wang *et al.*^31^ employed interpretable machine learning “sure independence screening and sparsifying operator” (SISSO), theoretical derivation, and first-principles calculations to demonstrate that MSI between metal nanoparticles (or single atoms) and oxide supports primarily consists of metal–metal (M–M′) and metal–oxygen (M–O) interactions (Fig. 2a–c). Their model identified the adsorption energy (*E*_adh_) as a key descriptor:

1 *E*_adh_ = *β*_1_ × *Q*(MO) + *β*_2_ × *Q*(MM′) + *β*_0_

where M denotes the element of supported metal and M′ refers to the metal in oxide, where *β*_0_ = 16 meV Å^−2^, 
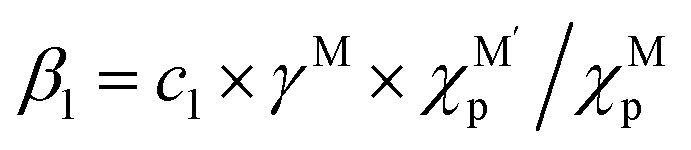
 and *c*_1_ = 9.85 × 10^−2^ eV^−1^. The ratio of Pauling electronegativity 
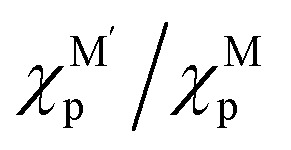
 shows the competitive bonding affinity of the two metal elements with oxygen, and systems with higher ratios are prone to having more interfacial M–O bonds, where 
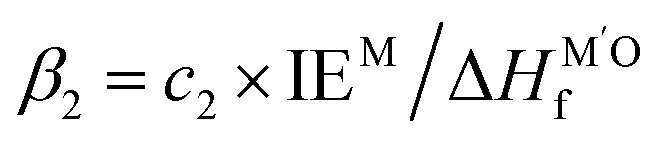
, *c*_2_ = −3.06 × 10^−3^ Å^2^, and 
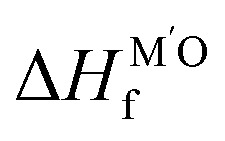
 is the formation enthalpy of oxide support.

This model was also applied to predict the contact angle of metals on oxide surfaces. Results indicate that optimal thermal stability is achieved when the MSI strength is moderate, corresponding to a contact angle near 90°, consistent with the findings of Liu *et al.*^32^ and Hu *et al.*^33^ Notably, encapsulation of nanoparticles within a support occurs when *Q*(MO) > *Q*(MM′). For single atoms, adsorption modes include M–O (Fig. 2a), M–M′ (Fig. 2b), and M′–M–O bonding (Fig. 2c), all of which can be quantified using the weighted combinations of *Q*(MO) and *Q*(MM′).

The strength of the M–O bond plays a critical role in stabilizing nanoparticles or single atoms. For instance, Pt nanoparticles are effectively stabilized on CeO_2_ under reducing or oxidizing conditions, whereas they sinter readily on SiO_2_—consistent with the conventional view of CeO_2_ as an active support and SiO_2_ as an inert one for Pt.^34^ However, this trend reverses for CoO_*x*_ nanoparticles: SiO_2_ strongly interacts with CoO_*x*_, retaining it in a low oxidation state (Co^2+^) even under CO_2_ hydrogenation conditions, while CeO_2_ binds CoO_*x*_ weakly, leading to its reduction to metallic Co^0^. Thus, for CoO_*x*_, SiO_2_ acts as an active support and CeO_2_ as an inert one. This behavior extends to other oxides such as MnO_*x*_ and NiO_*x*_, which are also more resistant to reduction on SiO_2_ than on CeO_2_. In contrast, Au follows the same trend as Pt, being more stable on CeO_2_ than on SiO_2_.

A general rule emerges: the stability of Pt correlates negatively with the O 1s binding energy (M–O bond strength) of the support, whereas that of CoO_*x*_ correlates positively. Supports with weak M–O bonds favor metal stabilization *via* strong MSI, while those with strong M–O bonds stabilize oxide species through strong oxide–support interactions. This work identified a linear trend between M–O bond strength and metal stability; however, the relationship was not quantitatively defined. Subsequently, Hu *et al.*^33^ developed a quantitative link between MSI and degradation mechanisms such as Ostwald ripening (OR) and particle migration and coalescence (PMC). The activation energy for OR was expressed in terms of binding energy (*E*_bs_) and cohesive energy (*E*_c_), while that for PMC involved *E*_bs_ and adhesion energy (*E*_adh_). These were normalized into a unified descriptor. Since OR and PMC occurred concurrently, and since *E*_adh_ and *E*_bs_ are positively correlated on a given support, the authors proposed a simplified relation:*E*_act_(OR) = 0.82*E*_bs_ − *E*_c_*E*_act_(PMC) = −*S*^m^*E*_adh_ − 0.33*E*_c_
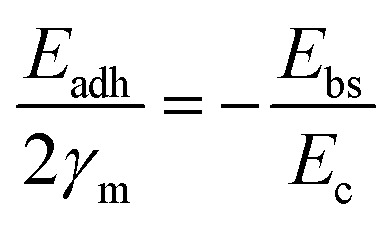


As shown in Fig. 2d, if the MSI is too strong, the contact angle becomes smaller. This improves the dispersion of metal nanoparticles and strengthens their binding to the support, which helps prevent migration and aggregation. However, it also makes OR more likely to occur. On the other hand, if MSI is too weak, the contact angle is large, and the nanoparticles form spherical shapes. In this case, PMC through van der Waals forces becomes easier, while atomic diffusion and OR are suppressed.

The best balance between activity and stability is achieved when MSI is moderate, which corresponds to a contact angle of around 90°. This idea is similar to the Sabatier principle in catalysis, which states that the interaction between a catalyst and a reactant should be neither too strong nor too weak. Similarly, MSI has an optimal range: if it is too strong, OR causes deactivation; if it is too weak, PMC leads to degradation. Both cases reduce the stability of the catalyst.

Shi *et al.*^35^ proposed the lowest unoccupied molecular orbital (LUMO) energy level of the support as another descriptor for SACs. Applying frontier orbital theory to MSI, they showed that as the support size decreases, the LUMO level rises, strengthening the interaction with the highest occupied molecular orbital (HOMO) of Pd single atoms and enhancing the stability (Fig. 2e). Additional studies suggest that the overlap area of density of states can also indicate MSI strength.

Notably, bonding between single atoms and support atoms follows molecular orbital principles, including symmetry matching and maximum overlap. In Cu–N–C structures, the σ bond formed between the Cu d_*x*^2^−*y*^2^_ orbital and the hybridized sp^2^ orbital of the N atoms shifts the energy of Cu d-orbitals.^36^ Two π bonds from d_*xz*_–p_*z*_ and d_*z*^2^_–p_*z*_ interactions raise the energy of orbitals with the *z*-axis component. Owing to the symmetry structure of M–N–C, d_*yz*_ behaves similarly to d_*xz*_, while non-*z* orbitals remain at a lower energy (Fig. 2f). The unique d^9^ electronic configuration of copper leads to the occupation of the d_*x*^2^−*y*^2^_ orbital at the Fermi level. This orbital plays the primary role in M–N bonding. The electrode potential will alter the Fermi level of the catalyst. Applying a more negative potential will raise the Fermi level. As the Fermi level gradually rises with the negative shift of the potential, it will align successively with the LUMO of the single atom sites and other frontier orbitals. Once the Fermi level aligns with or is higher than these orbitals, electrons will enter these orbitals from the electrode, thereby changing their electron occupation numbers. In the H–Cu–N–C system, particularly under reducing potentials, the transfer of electrons from the d_*z*^2^_ orbital to the d_*x*^2^−*y*^2^_ orbital induces a rearrangement of energy levels. Consequently, the d_*x*^2^−*y*^2^_ orbital forms a continuous energy band that spans the Fermi level (Fig. 2g). The adsorbed proton further enhances electron transfer into the antibonding component of the d_*x*^2^−*y*^2^_ orbital. This increased antibonding population weakens the Cu–N bond, resulting in displacement of the Cu atom from the C–N surface and a corresponding decrease in the stability of the SACs under reduction conditions.

In summary, molecular orbital theory offers a fundamental framework for understanding interactions between single atoms and their supports. Strong MSI requires energy level alignment, together with maximum orbital overlap during bonding. SAC stability follows a Sabatier-type principle, achieving an optimum at intermediate MSI strength. Under electrochemical conditions, electron transfer at varying potentials alters the occupancy of frontier orbitals, thereby affecting the SAC-support bond strength.

### 3.2 Descriptor governing the influence of MSI on the adsorption capability of SACs

The Sabatier principle states that the adsorption of reactants on a catalyst should be of moderate strength.^37^ Excessively weak adsorption impedes initial activation, while too strong adsorption stabilizes intermediates and hinders product desorption. Therefore, optimizing the adsorption energy is essential for enhancing the activity of SACs.

Zhou *et al.*^38^ classified the bonding interactions in SACs systems into three categories: (i) L–M_1_ coordination bond, between the single atom and the first-shell support atoms, governing the stability and oxidation state of the M_1_ site; (ii) A–M_1_ coordination bond, formed with adsorbates, enabling substrate capture and activation; (iii) L–support bond, connecting the first shell support atoms to the outer support framework, which constrains surface lattice atoms and modulates their reactivity.

Numerous studies have revealed a correlation between the valence state of metal atoms and their catalytic activity and stability;^39–41^ however, a direct and consistent relationship remains controversial. Molecular orbital theory, widely applied in homogeneous catalysis,^42^ attributes covalent bonding to the overlap of atomic or molecular orbitals, resulting in the formation of new molecular orbitals. Effective bonding requires three key conditions: energy alignment, symmetry matching, and maximum orbital overlap.^43,44^ In recent years, molecular orbital theory has been increasingly adopted as a fundamental descriptor in the study of SACs.^35,45,46^ A prevailing research focus is to optimize the adsorption energy and enhance the catalytic activity by modulating the MSI to influence the metal–adsorbate interaction (MAI).

The following section reviews descriptors that reflect how MSI influences the adsorption behavior of active sites, including the bonding triad principle, support effects, and electrostatic adsorption.

#### 3.2.1 Descriptors based on frontier molecular orbital theory

##### 3.2.1.1 Energy alignment

Greiner *et al.*^47^ proposed that single atoms in single-atom alloys (SAAs) exhibit a free-atom-like electronic structure. The Newns–Anderson–Grimley model was employed to describe the hybridization between adsorbate molecules and metal surface electrons. The model indicates that hybridization with a broad metal valence band (*e.g.*, s-orbital of Na) leads to broadening of the adsorbate state, while interaction with a narrow band (*e.g.*, d-orbital of Cu) causes splitting into bonding and antibonding states. Generally, a narrower band enhances the interaction strength. By performing a Hilbert transform of the d-band density of states and identifying its intersections with the adsorption function, one can distinguish between state splitting (multiple intersections) and broadening (single intersection).

**Fig. 3:**
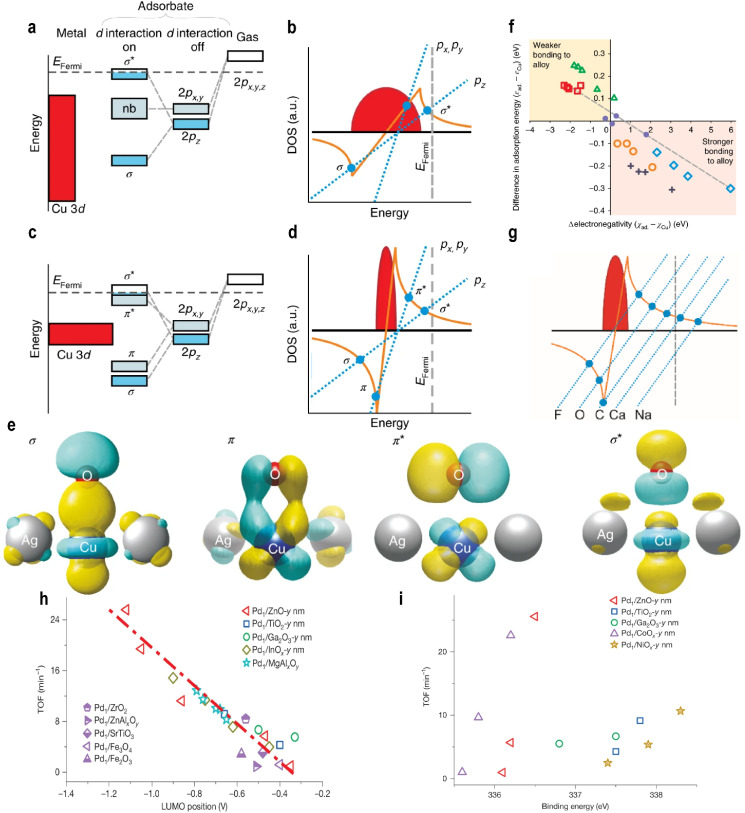
Schematic energy-level diagrams of the O 2p states as an O atom adsorbs to the Cu (a) and AgCu (c) surfaces. (b and d) Graphical solutions to the Newns–Anderson–Grimley model for O 2p_*z*_ and O 2p_*x*,*y*_ illustrating that the reason for the different bonding situations for the broad Cu 3d band and narrow Cu 3d states is the stronger interaction strength of the narrow d states. (e) The calculated wavefunctions illustrating the real-space bonding formed between O and Cu in AgCu. (f) The difference in adsorption energy between adsorbates on Cu_36_ and the Cu center of Ag_35_Cu_1_*versus* the adsorbate electronegativity (relative to the electronegativity of copper) for adsorbates. The symbols are colored according to the group and proceed from left to right in the order listed here. Red squares, group 1 (Cs, Rb, K, Na, and Li); green triangles, group 2 (Ba, Sr, Ca, Mg, and Be); purple dots, group 4 (Sn, Ge, Si, and C); orange circles, group 5 (Sb, As, P, and N); grey crosses, group 6 (Te, Se, S, and O); blue diamonds, group 7 (I, Br, Cl, and F). (g) A graphical solution to the Newns–Anderson model, illustrating how the orbital energy arises from the interaction between adsorbate states and the Cu 3d-band. Reproduced with permission.^47^ Copyright 2018, Springer Nature. (h) Linear scaling relationships between the activities of Pd_1_/MO_*x*_ catalysts and the LUMO positions of n-type. (i) The correlation between the TOFs of Pd/MO_*x*_ catalysts and the 3*d*_5/2_ binding energy of Pd_1_ atoms, as determined by *in situ* XPS. Reproduced with permission.^35^ Copyright 2025, Springer Nature.

For example, in the case of O adsorption on Cu_36_ (bulk catalyst), the adsorption line intersects the Hilbert transform once. Due to significant spatial overlap between O 2p_*z*_ and Cu 3d orbitals (with the *z*-axis normal to the surface), the O 2p_*z*_ orbital splits into bonding σ and antibonding σ* states. The O 2p_*x*_ and O 2p_*y*_ states do not bond with the Cu 3d band and remain nonbonding, resulting in broadening (Fig. 3a and b). On SAA, the narrow d-band couples strongly with oxygen states, yielding three intersections (Fig. 3c and d), indicating that the O p-orbitals and Cu d-orbitals satisfy both symmetry matching and a maximum spatial overlap. This interaction occurs not only between the O 2p_*z*_ and Cu d_*z*^2^_ orbitals but also involves the overlap of the Cu d_*xz*_ and d_*yz*_ orbitals with the O 2p_*x*_ and 2p_*y*_ orbitals (forming the σ bond), forming π bonds and antibonding π* states (Fig. 3e).

For a broader range of adsorbates, adsorption energy was found to correlate with the ionic and covalent character of the bond. Specifically, the electronegativity difference between the adsorbate and Cu serves as a key descriptor: adsorbates more electronegative than Cu bind more strongly to Ag_35_Cu_1_ than to Cu_36_, while those less electronegative bind more weakly (Fig. 3f). The adsorption behavior follows the principles of molecular orbital theory: energy level alignment, symmetry matching, and maximum orbital overlapping. Strong adsorption requires close energy proximity between interacting orbitals. For Group IA and Group IIA elements, their orbital energies lie far above the Fermi level of single atoms, leading to weak adsorption. In contrast, for Groups IV–VII, orbital energies are closer to the Fermi level, enabling stronger orbital splitting and adsorption (Fig. 3g).

A strong correlation exists between adsorption energy and electronegativity. For adsorbates within the same group, the adsorption energy difference scales with electronegativity (ionic character), while covalent interactions fine-tune the adsorption strength. Fundamentally, for elements in the same period (*e.g.*, B to F in period 2), the outermost p-orbitals have similar energies initially, but increasing nuclear charge and electron count along the period introduce shielding and penetration effects, progressively lowering the p-orbital energy and increasing the electronegativity. Thus, electronegativity partly reflects p-orbital energy. As p-orbital energy decreases from Group IV to VII, the energy gap with metal d-orbitals narrows, strengthening adsorption in accordance with the energy alignment principle (Fig. 3f).

This work offers important insights: parameters such as the charge on single atoms and the electronegativity of adsorbates, often associated with ionic or electrostatic effects, may fundamentally influence orbital energies. This suggests that a purely covalent model is insufficient. Therefore, the roles of electrostatic interactions and charge transfer in adsorption on single atoms warrant a deeper and more systematic investigation. In a subsequent study, Shi *et al.*^35^ examined how the support affects the frontier orbitals of single atoms and established a linear relationship between the frontier orbital energy levels of SACs and their catalytic activity (Fig. 3h). As the support size decreases, the LUMO energy of the support increases linearly with the turnover frequency (TOF) for acetylene semi-hydrogenation. *In situ* DRIFTS CO chemisorption showed a blue shift in the CO peak with decreasing support size, indicating a weakened Pd(4d)–CO(2π*) bond and increased electron deficiency of Pd atoms. This contradicts the classical charge theory, which associates the higher electron density with higher activity. The study revealed that charge-based descriptors are valid only within one type of support and fail across different supports (Fig. 3i). This limitation arises because the ionicity of the Pd–O bond is support-dependent but not size-sensitive, making charge an unreliable universal descriptor. DFT calculations showed that stronger Pd–support interaction increases electron transfer from Pd to the support, lowering the Pd LUMO energy and improving energy alignment with the HOMOs of acetylene and hydrogen, thereby promoting adsorption and H_2_ activation.

##### 3.2.1.2 Symmetrical matching

**Fig. 4:**
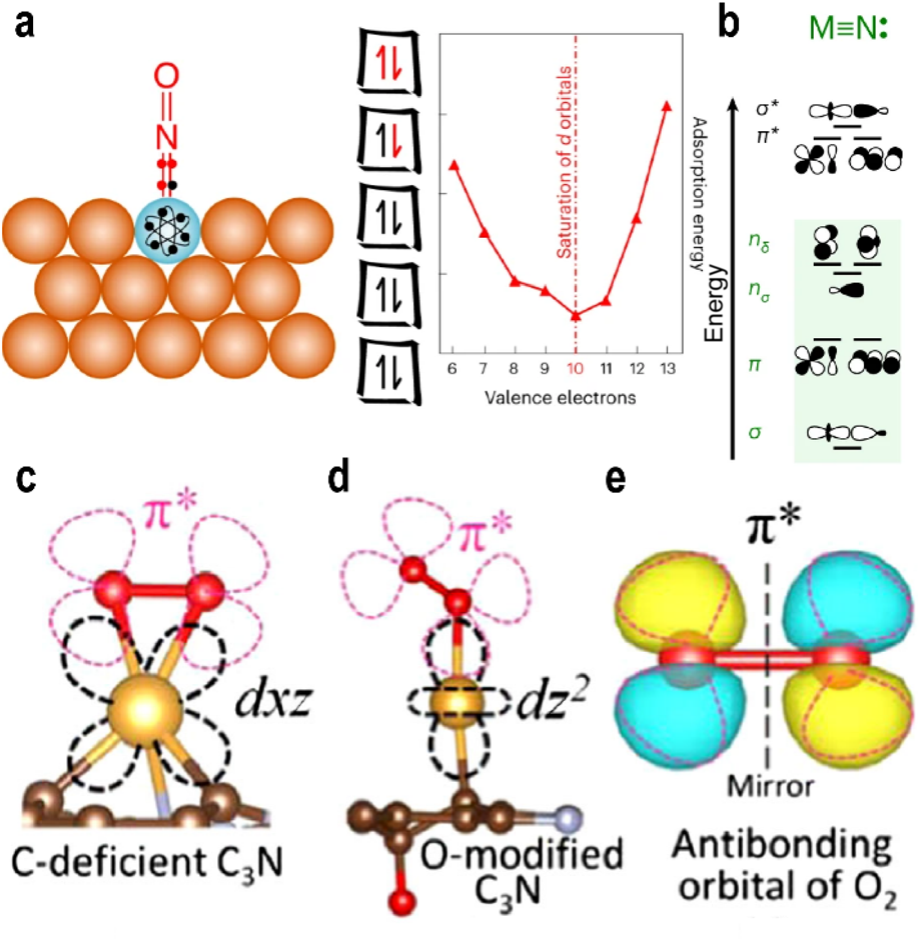
(a) Schematic diagram for the ten-electron count rule for the binding of adsorbates on Ag_35_Cu_1_ (SAA). (b) Molecular orbital diagrams for the dinuclear complexes MN. Reproduced with permission.^48^ Copyright 2024, Springer Nature. Different adsorption configurations on single-atom Au with two different frontier orbitals (c), for the adsorption of O_2_ (d), and the antibonding orbital of O_2_ (e). Reproduced with permission.^50^ Copyright 2020, American Physical Society.

In a 2024 study, Schumann *et al.*^48^ proposed the “10-electron count rule” for adsorption on SAAs from a molecular orbital perspective (Fig. 4a). This rule applies when adsorption is dominated by electron sharing (covalent contributions), and it accurately describes the adsorption energies of molecules such as CO and NO. Symmetry matching plays a critical role in the bonding between metal d-orbitals and adsorbate orbitals. The irreducible representations of metal d-orbitals are: a_1_(d_*z*^2^_), e_1_(d_*xz*_, d_*yz*_), e_2_(d_*xy*_, and d_*x*^2^−*y*^2^_). The valence orbitals of p-type adsorbates correspond to a_1_(s, p_*z*_) and e_1_(p_*x*_, p_*y*_). The metal d_*z*^2^_ orbital interacts with the adsorbate ss and p_*z*_ orbitals, generating three molecular orbitals: σ bond, σ* antibonding, and nσ nonbonding states. Similarly, the d_*xz*_ and d_*yz*_ orbitals combine in a linear combination manner with p_*x*_ and p_*y*_ orbitals to form two bonding π and two antibonding π* states (Fig. 4b).

In contrast, the d_*xy*_ and d_*x*^2^−*y*^2^_ orbitals lack matching symmetry with any adsorbate orbitals and thus remain nonbonding nδ.^49^ Electrons fill the five bonding and nonbonding orbitals before populating antibonding states. Adsorption strength is maximized when 10 electrons are occupied; beyond this point, further filling of antibonding orbitals weakens adsorption and facilitates adsorbate activation.

##### 3.2.1.3 Maximum orbital overlapping

The split orbital states of single-atom sites often lead to unconventional adsorption behavior, necessitating detailed investigation into their unique adsorption mechanisms.

In 2020, Fu *et al.*^50^ designed and computationally studied an Au^+^ atom supported on a carbon-defective substrate (Fig. 4c and d). They observed that the Au^+^ site exhibited higher activity than its Au^−^ counterpart, a finding that contradicts conventional charge-based interpretations. Compared to Au^−^ and Au^0^, the Au^+^ species exhibits stronger adsorption energy and a longer O–O bond length. Theoretical calculations revealed a maximal overlap between the d_*xz*_ orbital (HOMO) of Au^+^ and the 2π* orbital of O_2_, consistent with the three principles of molecular orbital theory: energy proximity, symmetry matching, and maximum overlap (Fig. 4c). The 2π* orbital extends into the Fermi level, enabling electron acquisition even when Au atom is in a positive oxidation state. This suggests that the ability to hybridize with the 2π* orbital, rather than the negative charge state, governs O_2_ activation and desorption. The authors further emphasized that the d-orbitals of Au split into discrete energy levels, the ordering of which is strongly influenced by the support. These findings underscore the critical role of the support in modulating the orbital characteristics of single atoms, indicating that variations in support properties can multi-dimensionally alter the orbital energy levels of the anchored metal species.

#### 3.2.2 Electrostatic adsorption

While frontier molecular orbital theory serves as a potential descriptor for SACs, alternative perspectives emphasize the significant role of electrostatic interactions. The 10-electron count rule having limits in describing the adsorption of molecules with lone pair electrons indicates that adsorption involves not only covalent bonding between adsorbates and SAC d-orbitals but also electrostatic attraction between lone pairs and metal sites.^48^ The essential distinction between covalent and electrostatic adsorption lies in the extent of electron transfer: covalent adsorption involves substantial electron sharing, while electrostatic adsorption, dominated by lone-pair interactions, entails minimal charge transfer.

Kakekhani *et al.*^51^ investigated electrostatic adsorption in species with lone pairs, highlighting the coexistence of covalent and noncovalent electrostatic effects during adsorption. Molecules such as H_2_O and NH_3_ possess dipole moments that induce image charges in the metal surface. The resulting induced dipoles further polarize the adsorbate and stabilize the lone-pair surface bond. In projected density of states (PDOS), this stabilization manifests as a gradual downward shift in the HOMO energy of H_2_O as it approaches the surface.

**Fig. 5:**
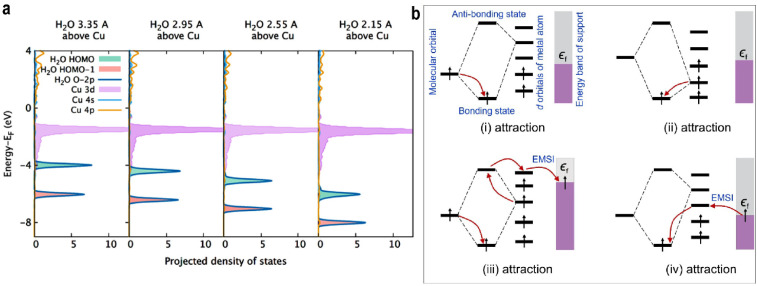
(a) PDOS of different distances of H_2_O from Cu@Au(111). Reproduced with permission.^51^ Copyright 2018, American Chemical Society. (b) The conceptual diagrams of electronic interaction between the O_2_ orbital, d orbital of SACs, and the support band for chemisorption. The black arrows indicate the electrons. Reproduced with permission.^57^ Copyright 2024, American Chemical Society.

On clean transition metal surfaces, the main contribution to bonding is covalent interaction (orbital hybridization and charge transfer). In contrast, adsorption on SACs exhibits a distinct mechanism where simple covalent models are limited. Intrinsic surface electrostatics (ISE), a noncovalent interaction, plays a dominant role. Due to electronegativity differences and low coordination numbers, Cu single atoms often carry a positive charge. Electron transfer from the less electronegative metal atom to more electronegative atoms (*e.g.*, O or N in adsorbates) results in a positive charge center surrounded by negative charges, generating a deep local electrostatic potential (ESP) well and a strong inhomogeneous surface electric field. This field stabilizes the HOMO of H_2_O along the *z*-direction, leading to the formation of an electrostatic bond. The fact that H_2_O can redistribute its electrons on the side facing the ESP well further stabilizes its bonding. The gradual energy downshift of the HOMO of H_2_O is evident in Fig. 5a. The study also confirmed that MSI modulates the charge on single atoms across different supports, thereby altering their electrostatic adsorption properties.

These findings collectively indicate that the coulombic force significantly influences the adsorption of oxygen species on SACs, with the charge of the metal playing a critical role. For example, Zhao *et al.*^52^ designed a curved Fe/N–C catalyst with a multilayer structure, in which electrostatic repulsion between nitrogen atoms in the outer layer and adsorbed oxygen intermediates in the inner layer weakened the binding strength of all oxygenated species, thereby enhancing oxygen reduction reaction (ORR) activity.

Nevertheless, research on electrostatic interactions in oxygen electrocatalysis remains limited. Further studies are essential to elucidate the relationship between the micro-environment of single atoms and their electrostatic adsorption properties, which may provide a viable pathway to break linear scaling relations. The adsorption of oxygen intermediates in the OER is fundamentally a synergistic process governed by electrostatic and covalent interactions. Initially, the intrinsically high oxidation state of the SACs generates a strong local ESP well. This ESP utilizes long-range coulombic attraction to interact with the lone pair electrons of the adsorbate molecule, achieving initial stabilization of the intermediate. This electrostatic contribution is quantified by descriptors like the Bader charge. After stabilization, the metal d-orbitals can engage in orbital hybridization with the adsorbate's orbitals, thereby forming the covalent bond. The essential role of MSI is to precisely tune the electronic occupancy of the d-orbitals, which in turn fine-tunes the strength of the covalent interaction. This fine-tuning is crucial as it allows adsorption energy to be optimally adjusted to the peak of the Sabatier curve, thus circumventing the limitations imposed by linear scaling relationships. The local electric fields on high-curvature carriers or nanoneedles can help to understand this theory. Wang *et al.*^53^ synthesized Mn single atom doped CoP nanoneedles; the strong local electric field at the nanoscale can cause more OH^−^ to accumulate near the electrode surface, while the electronic interaction at Mn sites promotes the desorption of O*. The local electric field on the high-curvature surface can also optimize the adsorption configuration of water molecules and increase the concentration of the reactant near the surface.^54–56^

#### 3.2.3 Support effect

During adsorption, EMSI enables electrons from the support to participate in bonding, minimizing the total electronic energy of the adsorbate-single atom-support system.^57^ Notably, even Sc^3+^, with its entirely empty d-orbitals, can induce a negatively charged O_2_ species, indicating that electron donation originates from the support. This electron transfer from the support to adsorbed O_2_ is likely mediated by EMSI during chemisorption. The electron filling behavior is summarized as Fig. 5b:

(i) If the adsorbate molecular orbital is occupied and the interacting SAC orbital is unoccupied, electrons from the adsorbate fill bonding orbitals, lowering the system energy.

(ii) If the SAC orbital is occupied and the adsorbate orbital is unoccupied, electrons from the SAC fill bonding orbitals, stabilizing the adsorbate.

(iii) If both the adsorbate and SAC orbitals are occupied with parallel spins, electrons from the adsorbate occupy bonding orbitals, while electrons from the SAC enter antibonding orbitals and are further transferred *via* the single atom to lower-energy states in the support.

(iv) If both orbitals are unoccupied, electrons would still be transferred from the support into the bonding orbitals through the single atom, reducing the total energy and stabilizing the adsorbate.

Through EMSI, this process alters the electron distribution between the SAC and the adsorbate. The support acts as an electron sea, accepting or donating electrons to reduce the overall electronic energy, thereby modulating adsorption strength.

## 4. Mechanism

Understanding the strength of MSI is crucial for predicting the adsorption behavior of the active sites in SACs, which we now explore through specific descriptors. These descriptors help to connect the fundamental interactions with the observed catalytic behaviors, guiding the identification of rate-determining steps and reaction pathways. In the electrochemical process, the applied potential can shift the Fermi level of the catalyst. At the reaction potential, electrons transfer between the frontier orbitals of the catalyst and the external circuit.^58^ Therefore, frontier molecular orbital theory remains applicable in single-atom electrocatalysis.

The reaction rate *P*, governed by such localized interactions, is proportional to the overlap integral of the two orbitals and inversely proportional to their energy difference. This relationship can be expressed as 
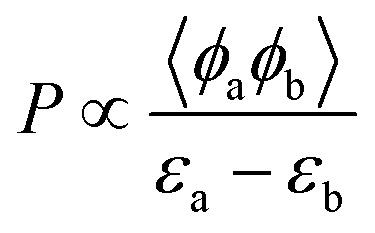
, where 〈*ϕ*_a_|*ϕ*_b_〉 is the overlap integral and *ε*_a_ − *ε*_b_ is the energy difference between the interacting orbitals.

When the orbitals of the SAC and the reactant are energy-aligned and symmetry-matched, electron transfer occurs efficiently. Thus, frontier molecular orbital theory provides a useful framework for evaluating the feasibility of electron transfer in electrocatalytic systems.

The OER is a fundamental electrochemical process central to many clean energy technologies. As the anodic half-reaction in water splitting, the OER suffers from slow kinetics and high overpotential, representing a major bottleneck for the overall efficiency. In SACs, MSI can modulate the electronic structure of both the single atom and the support, thereby tuning the adsorption energy of reaction intermediates, enhancing the OER activity, and even altering the reaction mechanism. Current OER mechanisms include the conventional adsorbate evolution mechanism (AEM), as well as alternative pathways that circumvent the scaling relations limiting the AEM, such as the oxide path mechanism (OPM) and the lattice oxygen mechanism (LOM). A deeper understanding of these pathways is essential for designing highly efficient and stable SACs.

### 4.1 Adsorbate evolution mechanism (AEM)

**Fig. 6:**
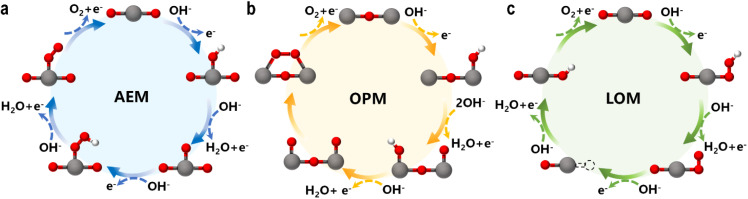
Schematics of OER mechanisms: the (a) adsorbate evolution mechanism (AEM), (b) oxide path mechanism (OPM), and (c) lattice oxygen mechanism (LOM).

The AEM is a typical OER pathway. In this mechanism, the reaction proceeds entirely through adsorbed oxygen intermediates (OH*, O*, and OOH*) formed sequentially on the active site.^59^ In alkaline media, the steps are as shown in Fig. 6a:OH^−^ + * → OH* + e^−^OH* + OH^−^ → O* + H_2_O + e^−^O* + OH^−^ → OOH* + e^−^
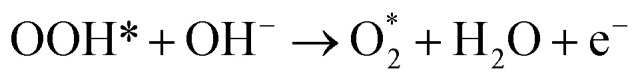

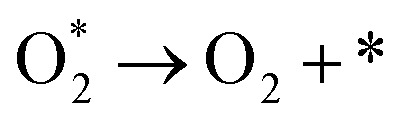


In acidic media, the reaction begins with water adsorption and proceeds through deprotonation steps.H_2_O + * → OH* + H^+^ + e^−^OH* → O* + H^+^ + e^−^O* + H_2_O → OOH* + H^+^ + e^−^OOH* → O_2_ + H^+^ + e^−^ + *

A key limitation of AEM is the linear scaling relation between the adsorption energies of OH* and OOH*, which imposes a minimum theoretical overpotential.^60^

### 4.2 Oxide path mechanism (OPM)

The OPM can occur at single or dual sites. For the dual-site pathway, a suitable M–M distance is critical to facilitate oxygen bond formation.^61^ In the OPM, two OH* species deprotonate to form O* intermediates, which then couple to form O_2_, bypassing the unstable OOH* intermediate and oxygen vacancy formation (Fig. 6b). This pathway often enhances catalyst stability.2OH^−^ + 2* → 2OH* + 2e^−^2OH* + 2OH^−^ → 2O* + 2H_2_O + 2e^−^2O* → O* − O*O* − O* → O_2_ + 2*

### 4.3 Lattice oxygen mechanism (LOM)

The LOM is a novel pathway increasingly identified in high-performance oxide-supported SACs (*e.g.*, on IrO_2_, MnO_2_, RuO_2_, *etc*.).^62–64^ In Fig. 6c, the O* intermediate formed from OH* deprotonation directly couples with a lattice oxygen (O_L_) to form an O–O bond, releasing O_2_. This mechanism involves the participation and subsequent regeneration of lattice oxygen.OH^−^ + O_L_ → O_L_OH* + e^−^O_L_OH* + OH^−^ → O_2_ + V_O_ + H_2_O + e^−^V_O_ + OH^−^ → O_L_H + e^−^O_L_H + OH^−^ → O_L_ + H_2_O + e^−^

Studies have shown that during the OER process, the “*z*-axis” orbitals of SACs play a primary role. In an octahedral ligand field, the e_g_ orbitals consist of the d_*z*^2^_ and d_*x*^2^−*y*^2^_ orbitals.^65^ As previously discussed, the d_*z*^2^_ orbital primarily interacts with adsorbates, while the d_*x*^2^−*y*^2^_ orbital engages mainly with the support.^36^ Thus, the e_g_ orbitals collectively govern both the MSI and the MAI. Consequently, the electron occupancy of the e_g_ or “*z*-axis” orbitals may serve as a key descriptor for OER activity. Suntivich *et al.*^66^ identified a volcano-shaped correlation between OER activity and the number of electrons in the e_g_ orbitals across various perovskite oxide electrocatalysts, with peak activity predicted at an occupancy of one. This emphasizes the significance of the spin configuration in relation to the adsorption energy, rather than barely on the oxidation state or charge-based models.

In the OER, oxygen species also adsorb *via* electrostatic interactions with lone-pair electrons, which limits the molecular orbital theory to fully and accurately describe the adsorption of oxygen species on SACs. Therefore, current descriptors for oxygen adsorption in the OER are adopted from both frontier molecular orbital theory and charge-based models. A more accurate descriptor should integrate the symmetry matching, maximum orbital overlap, and energy alignment from molecular orbital theory, together with electrostatic contributions from charge theory, with appropriate weighting.

Recently, Xu *et al.*^67^ employed a progressive learning model to predict the OER overpotential (*η*_OER_) for 261 M_SA_–MO_*x*_ systems and applied subgroup discovery (SGD) to identify key descriptors. They found that high OER activity is associated with surface metal Bader charge qSMs ≤ −1.32*e*, surface oxygen p-band center pOs ≥ −2.83 eV, and doped metal HOMO energy Hm ≤ −3.22 eV.

A qSMs value ≤−1.32*e* suggests a highly oxidized support, facilitating charge transfer and improving electrostatic adsorption capability. A pOs ≥ −2.83 eV indicates strong hybridization between the oxygen p-band and metal d-states, reflecting strong MSI. Hm ≤ −3.22 eV corresponds to low HOMO energy and strong MAI. This work highlights the critical role of both the frontier orbital energies and electrostatic adsorption properties of SACs in determining the adsorption energies of OER intermediates.

This study serves as an example for OER descriptor development, incorporating both the oxidation state of the catalyst and the frontier orbital energy of the single atom while highlighting the critical role of the support in tuning the adsorption properties of SACs.

Descriptors of MSI also reflect the selectivity of the OER pathway by quantifying the electronic properties of SACs. A high oxidation state of the metal center weakens the metal–oxygen covalency, reduces the adsorption strength of the O* intermediate, and thereby promotes the AEM while suppressing O_L_ participation.^67^ In contrast, strong metal–oxygen covalency and adsorption facilitate the LOM. MSI can elevate the p-band center of adjacent oxygen atoms closer to the Fermi level, thereby facilitating O_L_ participation. The OPM becomes accessible when two metal active sites are positioned at a suitable distance (3 Å) and both contribute to the HOMO of the catalyst.^68,69^ From a chemical perspective, coordination ligands modulate the splitting of d-orbitals, influencing intermediate stability and electron transfer pathways, which collectively govern the mechanistic selectivity.

## 5. Design strategies and applications of MSI in SACs for the OER

The catalytic performance of SACs can be effectively predicted using a series of descriptors, which reveal that the adsorption behavior at the active site is a complex process governed by both covalent and electrostatic interactions. On one hand, the interaction between the catalyst and reactants follows frontier molecular orbital theory, where chemical bonds form through effective overlap between the metal d-orbitals and the molecular orbitals of adsorbates, thereby activating the reactants. On the other hand, electrostatic attraction (coulombic interaction) plays a critical role in the adsorption of oxygen species containing lone-pair electrons, which are common in the OER. Therefore, an ideal active site requires synergistic optimization of its electronic structure (*e.g.*, d-band center, frontier orbital energy, *etc*.) and charge state (*e.g.*, Bader charge, oxidation state, *etc*.) to achieve moderate adsorption strength. This section introduces catalyst design strategies based on spin configuration, axial coordination, atomic distance, spatial position, and support defects. These methods modulate the local coordination environment of the single atom through MSI, thereby enhancing catalytic activity.

### 5.1 Spin configuration

Guided by molecular orbital theory, researchers have utilized MSI to tune the electronic state of SACs, optimizing the adsorption energy of reaction intermediates and improving OER activity. During this process, adsorbates typically form bonds through hybridization with the d orbitals of the active metal site. The electron occupancy and spin orientation in these d orbitals directly influence the bond strength, which in turn governs molecular adsorption/desorption behavior and electron transfer kinetics. Spin direction modulation can also lower reaction energy barriers. In the OER, reactants such as OH^−^ and H_2_O are singlet species, while the product oxygen has a triplet ground state with parallel spin alignment (↑O = O↑). The transition from singlet intermediates to triplet oxygen is spin-forbidden. It has been reported that the energy of triplet oxygen is approximately 1 eV lower than the singlet state.^70^ When the active sites exhibit highly polarized parallel spins, the electrons they provide can more readily form spin-parallel O–O bonds, converting the reaction pathway from “spin-forbidden” to “spin-allowed” and reducing the energy barrier. This explains why external magnetic fields can enhance OER activity. Thus, spin regulation in SACs is an important consideration in catalyst design.^71,72^ Elements such as Fe, Co, and Ni, which are inherently ferromagnetic, have attracted significant attention in this context.^72–74^

**Fig. 7:**
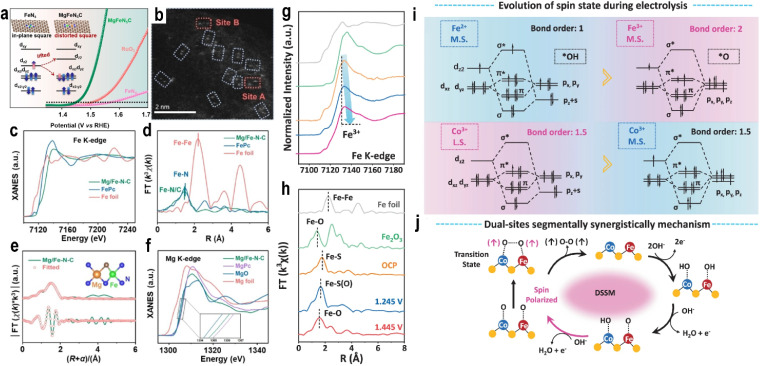
(a) Schematic for improving electrocatalytic oxygen evolution through local field distortion in Mg/Fe dual-site catalysts. (b) HAADF-STEM image (dual-sites are highlighted by dotted lines). (c) Fe K-edge XANES spectra. (d) Fe K-edge Fourier transform of the EXAFS spectra in R space. (e) Fe K-edge EXAFS fitting curves of Mg/Fe–N–C; (inset) proposed architecture of Mg–Fe–N_5_–C_1_. (f) Mg K-edge XANES spectra. Reproduced with permission.^75^ Copyright 2023, Wiley-VCH. (g) Fe K-edge XANES spectra. (h) Fe K-edge FT-EXAFS during the OER process with different potentials (*vs.* RHE) of OCV, 1.245 V, and 1.445 V in 1 M KOH, respectively. (i) The orbital interactions between cations and the OER intermediates of Fe and Co during the OER process according to the bond order theorem. The red region represents more favorable metal spin–orbit coupling interactions. (j) The schematic diagram of dual-site adsorbate evolution mechanisms for coupled O–O bonding during the OER (blue for Co atoms, red for Fe atoms, and yellow for S atoms). Reproduced with permission.^76^ Copyright 2024, Springer Nature.

The rearrangement of d-orbitals may cause a change in the relative values between the crystal field splitting energy and the spin pairing energy, thereby leading to a change in the spin state. In the local structure surrounding the central metal atom, the substitution of heteroatoms can regulate the adjacent coordination environment of the metal, causing the coordination field distortion and changing the spin state. Zhang *et al.*^75^ synthesized a dual-atom Mg/Fe–N–C catalyst *via* a one-step wet chemical method. The introduction of the main-group Mg atom enhanced crystal field distortion and modified the spin state of the Fe atom (Fig. 7a). Aberration-corrected high-angle annular dark-field scanning TEM (HAADF-STEM) images clearly revealed dual-atom sites (Fig. 7b). X-ray absorption near-edge spectroscopy (XANES) spectra in Fig. 7c at the Fe K-edge indicated an average oxidation state close to +2 for Fe in Mg/Fe–N–C, similar to FePc but distinct from Fe foil, and a deviation from the ideal *D*_4h_ symmetry. FT-EXAFS analysis showed a dominant peak at 1.50 Å, corresponding to Fe–N/Fe–C interactions, with a slightly lower intensity than FePc, suggesting a reduced coordination number (Fig. 7d). Wavelet transform analysis and fitting results confirmed coordination numbers of 3.2 for N and 0.9 for C (Fig. 7e). Mg K-edge XANES spectra in Fig. 7f further verified the atomic dispersion of both Fe and Mg. In OER performance, Mg/Fe–N–C exhibited a low onset potential of 1.40 V, superior to commercial RuO_2_ (1.47 V), Fe–N–C (1.52 V), and Mg–N–C (1.54 V).

The large ionic radii mismatch between Mg^2+^ and Fe^2+^ can cause a FeN_4_ in-plane square local field deformation, raising the energy of the d_*z*^2^_ orbital and causing electrons to redistribute to d_*xy*_/d_*yz*_, which triggers a favorable spin transition of Fe^2+^ from intermediate spin (IS) (d*_xy_*^2^d*_xz_*^2^d*_yz_*^1^d_z^2^_^1^, 2.96*µ*_B_) to low spin (LS) (d_xy_^2^d_xz_^2^d_yz_^2^, 0.95*µ*_B_) and enabling overlap between the “*z*-axis” frontier orbitals of Fe and the O 2p_*z*_/2p_*y*_ and π* orbitals of O*/OH* and OOH* along the *z*-direction. The subsequent depopulation of the d_*z*^2^_ orbital reduces orbital overlap along the *z*-axis, weakening adsorption strength and optimizing catalytic activity. At a current density of 10 mA cm^−2^, Mg/Fe–N–C exhibited an overpotential of 224 mV, which was 123 mV and 163 mV lower than that of Fe–N–C and Mg–N–C, respectively (Fig. 7a).

Xu *et al.*^76^ synthesized a Fe, Co dual-atom catalyst (CFS-ACs/CNT) *via* an adsorption–reduction hydrothermal method. XANES confirmed the atomic dispersion of Fe and Co. Magnetic characterization, ^57^Fe Mössbauer spectroscopy, and XANES analysis revealed distinct magnetic states: Fe^2+^ existed in an intermediate-spin state (t^5^_2g_e^1^_g_), while Co^3+^ was in a low-spin state (t^6^_2g_e^0^_g_). CFS-ACs/CNT demonstrated exceptional OER activity, with an overpotential of 270 mV at 20 mA cm^−2^, outperforming commercial IrO_2_ and FeS_*x*_/CNTs by 100 mV and 80 mV, respectively. *In situ* XANES spectra showed that Fe^2+^ was gradually oxidized to Fe^3+^ during the OER (Fig. 7g). As the applied potential increased from 1.245 V to 1.445 V, the main peak shifted toward lower energy, indicating changes in MSI and the Fe–S coordination environment (Fig. 7h). Both Fe and Co undergo spin state changes during the reaction process (Fig. 7i). Fe^3+^ in an intermediate-spin (t^4^_2g_e^1^_g_) exhibited strong O* adsorption energy, lowering the O–O coupling barrier. OH^−^ preferentially adsorbed on the Fe site, followed by deprotonation to form O*, triggering a dual-site OPM (Fig. 7j). The different spin states of the two atoms in the dual-atom catalyst facilitate the adsorption of distinct intermediates, breaking linear scaling relations. Li *et al.*^77^ prepared a catalyst with atomically dispersed Ce on CoO. The Ce–O–Co bond created an electronic energy gradient, enhancing the Co–O covalency and catalytic stability. PDOS analysis revealed that the electrons in Co d orbitals changed from e^2^_g_ to spin-down e^1^_g_. The interaction between spin-down d_*yz*_ and d_*x*^2^−*y*^2^_ orbitals of Co and spin-up electrons of oxygen species facilitated the formation of triplet O_2_ σ and π bonds. Sun *et al.*^78^ prepared a Ni SAC on MoS_2_ (Ni_1_/MoS_2_) with a distorted quadrilateral structure, inducing ferromagnetic coupling with adjacent S atoms and neighboring Ni sites. *In situ* characterization and theoretical calculations identified the non-bonded S_1_ atom adjacent to the Ni site as the true active site for the OER. This ferromagnetic coupling favored spin-selective charge transfer, enabling triplet O_2_ generation. This also illustrates that SACs are not always single-site catalysts, and the support plays a vital role, demonstrating the significant influence of MSI.

Modulating the spin state of SACs *via* MSI is a powerful strategy to enhance the catalytic performance. For the OER, this approach overcomes the spin barrier between singlet reactants and the triplet oxygen product. Engineering dual-atom sites or specific supports alters the active metal's electronic structure, which lowers the energy barrier for this spin-forbidden step. This directly accelerates the reaction rate and boosts the overall efficiency.

### 5.2 Axial coordination

The electronic state of a single atom is directly influenced by its first-shell coordination environment, and axial coordination is an effective strategy for modulating the spin state of the metal center.^79^ Atoms on the support (*e.g.*, O, N, C, *etc*.) provide anchoring sites for the metal center.^80^ The type and electronic state of the coordinating atoms determine the charge transfer between the single atom and the support, directly affecting the splitting pattern and magnitude of the d-orbital energy levels, thereby altering the spin state of the single atom (HS ⇆ IS ⇆ LS). In an octahedral crystal field, the d-electrons of the central atom occupy the t_2g_ (d_*xy*_, d_*yz*_, d_*xz*_) and 
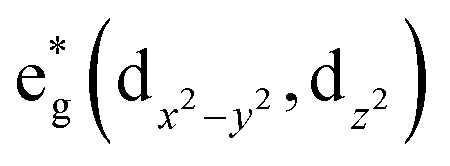
 orbital sets. The relative magnitudes of the electron pairing energy (*P*) and the splitting energy (*Δ*_o_) determine the spin state: weak field ligands (*P* > *Δ*_o_) favor high spin (HS) complexes with maximum unpaired electrons, while strong field ligands (*P* < *Δ*_o_) favor LS complexes with paired electrons in the lower-energy t_2g_ orbitals.

Spin-state-dependent ionic radius changes are significant in biochemistry. For example, in hemoglobin, the Fe^2+^ in the heme cofactor reversibly binds oxygen. In the oxygenated state, Fe^2+^ is low-spin with a smaller radius, allowing it to fit into the porphyrin plane as a six-coordinate complex. Upon deoxygenation, Fe^2+^ becomes high-spin with a larger radius, resulting in a five-coordinate structure where the metal is displaced from the porphyrin plane. Numerous studies have explored the design and application of the axial coordination ligand for SACs. Pan *et al.*^81^ developed a Co–N_5_ catalyst with axial N coordination as a robust electrocatalyst for CO_2_ reduction, exhibiting nearly 100% CO selectivity and remarkable stability.

Qiao *et al.*^82^ reported a Fe–N–C SAC featuring axial nitrogen coordination. By modulating the axial ligand from the weak-field chlorine to the strong-field nitrogen, the spin state of Fe was tuned from HS to IS. This spin-state transition optimized the binding strength of OH*, thereby enhancing the bifunctional activity of the N-FeN_4_ site for both the OER and ORR. DFT calculations revealed that the spin-state transition strengthened the interaction between the Fe site and OH*, significantly lowering the OER energy barrier.

**Fig. 8:**
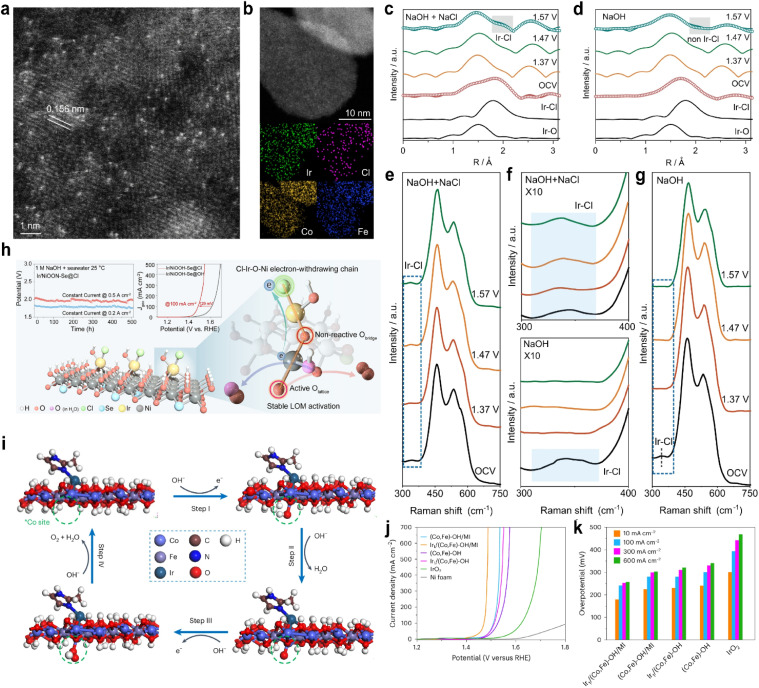
(a) HAADF-STEM image of Ir/CoFe-LDH showing single atomic Ir dispersion on CoFe-LDH. (b) Dark-field TEM image of Ir/CoFe-LDH and the corresponding elemental mappings showing the distribution of Fe, Co, Cl, and Ir elements. (c) *In situ* Fourier-transformed EXAFS spectra of Ir/CoFe-LDH in (c) NaOH + NaCl and (d) NaOH. *In situ* Raman spectra of Ir/CoFe-LDH recorded in (e) NaOH + NaCl and (g) NaOH. (f) Enlarged views of (e) and (g); the blue-highlighted portions correspond to the Ir–Cl coordination. Reproduced with permission.^83^ Copyright 2024, Springer Nature. (h) Schematic of the lattice oxygen mechanism induced on nickel sites by Cl^−^ adsorption for an efficient seawater oxidation reaction. Reproduced with permission.^84^ Copyright 2025, American Chemical Society. (i) Proposed 4e^−^ mechanism of the OER at the Co site adjacent to Ir in Ir_1_/(Co,Fe)-OH/MI. (j) LSV curves in 1 M KOH at a scanning rate of 5 mV s^−1^. (k) Overpotentials at 10 mA cm^−2^, 100 mA cm^−2^, 300 mA cm^−2^ and 600 mA cm^−2^. Reproduced with permission.^88^ Copyright 2024, Springer Nature.

Because seawater contains abundant chloride ions, SACs with axial coordination of chlorine have been widely reported in seawater electrolysis. Duan *et al.*^83^ developed an Ir SAC supported on CoFe-layered double hydroxide (Ir/CoFe-LDH) to modulate chloride adsorption and regulate the electronic structure of the Ir active center. The HAADF-STEM image (Fig. 8a and b) and XANES and EXAFS spectra confirmed the atomic dispersion of Ir on the surface. In 6 M NaOH + 2.8 M NaCl electrolyte, Ir/CoFe-LDH exhibited superior OER performance with an overpotential of 202 mV and a TOF of 7.46 O_2_ s^−1^, outperforming the system without Cl^−^ (overpotential 236 mV; TOF = 1.05 O_2_ s^−1^). In the *operando* EXAFS spectra, only Ir–O could be observed in NaOH (Fig. 8c), while both Ir–Cl and Ir–O were present simultaneously in the NaOH + NaCl electrolyte (Fig. 8d). *In situ* Raman analysis consistently showed Ir–Cl at 333 cm^−1^ in Cl^−^-containing electrolyte, while only a weak signal was observed at open-circuit voltage in Cl^−^-free electrolyte (Fig. 8e–g), indicating competitive adsorption between Cl^−^ and OH^−^ at high OH^−^ concentrations. DFT identified the formation of OOH* as the rate-determining step. Crystal orbital Hamiltonian population (COHP) analysis revealed stronger bonding interactions between the molecular orbitals of OOH* and the Ir site in the Ir–Cl–OH configuration compared to Ir–Cl–Cl or Ir–OH–OH, suggesting enhanced OOH adsorption on Ir–Cl–OH and a lower energy barrier for the rate-determining step. Furthermore, the hybridization between Ir and Cl bands was significantly enhanced. The strong Ir–Cl coordination stabilized Co and Fe sites, suppressed Cl^−^ coordination to these metal centers, and ensured stability during seawater electrolysis, retaining operation for over 1000 hours at high current densities. Sha *et al.*^84^ synthesized an Ir SAC with axial Cl^−^ coordination (Ir/NiOOH-Se@Cl). The strong electron-withdrawing effect of Cl^−^ established a Cl–Ir–O–Ni electron-withdrawing chain (Fig. 8h), which enhanced Ni–O covalency and promoted lattice oxygen participation, shifting the reaction mechanism from the AEM to the LOM. *In situ* Raman spectroscopy confirmed the stability of the Ir–Cl coordination structure in seawater electrolyte, along with a significantly higher Ni^4+^ content compared to that under alkaline conditions. Isotope labeling and differential electrochemical mass spectrometry (DEMS) jointly verified the involvement of lattice oxygen. In 20 wt% NaOH + saturated NaCl, the Ir/NiOOH-Se@Cl catalyst required an overpotential of only 313 mV at 0.5 A cm^−2^, 147 mV lower than that in alkaline electrolyte and remained stable for over 500 hours.

Axial Cl^−^ coordination has also been applied to enhance ORR activity. For instance, the relatively inert main-group element Sb exhibited exceptional ORR performance when axially coordinated by Cl^−^, achieving a half-wave potential of 0.92 V with only an 11 mV decay after 5000 cycles. Mechanistic studies indicated that Cl^−^ coordination precisely modulates the p-orbital electron density of the central Sb atom, lowering the OH* adsorption energy and improving the ORR activity.^85^ Similarly, Cl^−^ effectively regulates noble metal single atoms; a Pt_1_Co_1_/NC-Cl dual-atom catalyst with axial Cl^−^ coordination achieved a half-wave potential of 0.84 V, with only a 12 mV loss after 5000 cycles. DFT calculations revealed that axial Cl coordination modulates the electronic structure of Pt, reducing the OOH* adsorption energy barrier and enhancing the ORR performance.^86^

Chlorine, with its high electronegativity and large ionic radius, optimizes the orbital energy distribution of metal centers, leading to optimal adsorption energy for oxygenated intermediates. Moreover, the p-orbitals of Cl can form π-backbonding with metal d-orbitals, resulting in strong interactions that stabilize single atoms. This unique regulation makes Cl^−^ a promising axial ligand, successfully applied in the ORR, OER, and CO_2_ reduction reaction (CO_2_RR).^81^

Organic molecules have also been employed as axial ligands in SACs. For example, introducing axial ligands (CH_3_/CN) into pyrrole-type CoN_4_ sites was shown to enhance the OER/ORR activity.^87^ Larger organic axial ligands have also been reported; Zhao *et al.*^88^ prepared an Ir SAC coordinated with dimethylimidazole (MI) on CoFe hydroxide nanosheets. The Ir_1_/(Co,Fe)-OH/MI catalyst exhibited an ultralow overpotential of 179 mV at 10 mA cm^−2^ (Fig. 8j and k). First-principles calculation attributed this exceptional OER performance to the unique coordination between the Ir single atom and MI. Axial MI coordination induced effective charge redistribution around the Ir site, influencing not only the Ir atom but also adjacent Co atoms, resulting in favorable shifts in the d-band centers of both Ir and Co. The neighboring Co site exhibited an overall energy barrier of only 0.78 eV, identifying Co as the actual active site (Fig. 8i).

Chen *et al.*^89^ reported a CoN_4_ site by axial coordination of Co–S (Co_1_N_4_-S_1_) for the ORR. The optimal Co_1_N_4_-S_1_ exhibits excellent alkaline ORR activity, according to the half-wave potential (0.897 V) and Tafel slope (24.67 mV dec^−1^). Moreover, the Co_1_N_4_-S_1_ based Zn–air battery displays a high power density of 187.55 mW cm^−2^ and an outstanding cycling stability for 160 h. DFT calculations indicated more occupied π* orbitals of oxygen species on OOH@Co_1_N_4_-S_1_ and thus make it easier to break the O–O bond. This indicates that there is a great research potential for the development of axial ligands.

Traditional SACs design has largely focused on the two-dimensional plane of the coordination environment. Axial coordination engineering introduces a three-dimensional perspective into catalyst design. This strategy allows precise installation of specific functional ligands at the atomic scale, regulating MSI and bringing about significant changes to the electronic and catalytic properties of single atoms.

### 5.3 Atomic distance

Traditional models of SACs typically emphasize the isolated nature of metal sites, where active centers are sufficiently separated to prevent aggregation. Recent advances have revealed that precisely controlling the distance between single-atom sites, or tuning their surface density, offers a promising strategy for enhancing catalytic performance.^90–92^ When the interatomic distance is regulated within a specific range, it gives rise to distinct single atom–support interactions or single atom–single atom interactions. These effects subsequently modify orbital hybridization and spin coupling between adjacent sites, thereby enabling cooperative adsorption and evolution of intermediates.

Tang *et al.*^93^ demonstrated that the density of single atoms influences their OER activity by modulating the adsorption energy of OH*. The OER activity followed the increasing order: 1-CuN_3_ < 2-CuN_3_ < 3-CuN_3_ < C_3_N_3_Cu. Notably, isolated or low-density Cu SACs exhibited minimal OER activity, confirming that the performance enhancement is governed by electronic structure modifications resulting from higher atomic density rather than simply from an increased number of active sites. Kumar *et al.*^94^ proposed a high-density Co SAC (10.6 wt%) in a pyridine-rich graphene network. *Operando* XANES analysis identified the formation of an electron-deficient Co–O intermediate, enhancing the OER activity. Similarly, Song *et al.*^95^ combined theoretical and experimental approaches to show that the catalytic activity for benzene hydroxylation is proportional to the density of Cu single atoms (0.1–2.4 atoms per nm^2^). Studies revealed that interactions between adjacent SACs also modify their electronic structures, indicating that increased SAC density not only raises the number of active sites but also significantly modifies the electronic properties of metal centers.^96^

**Fig. 9:**
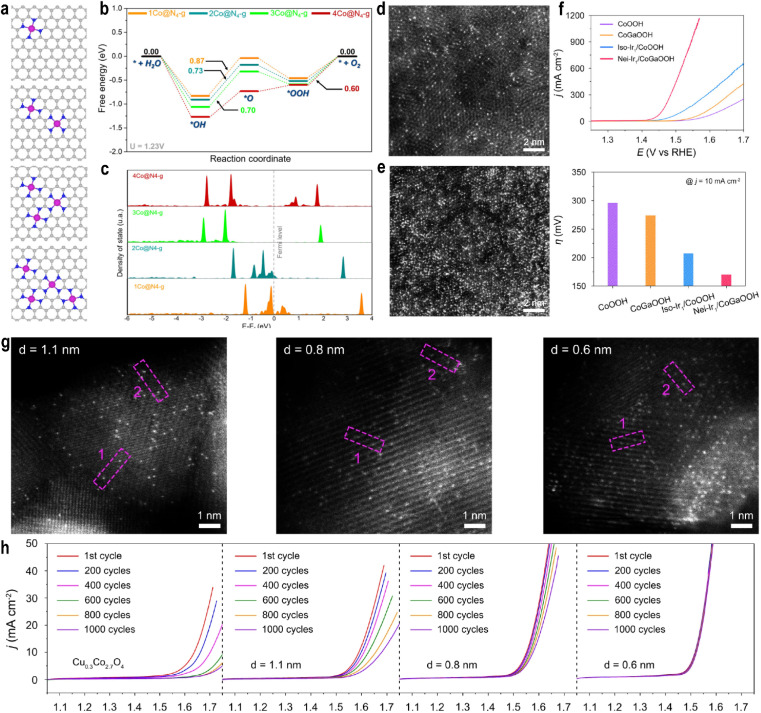
(a) Optimized structure of Co@N_4_-g at different densities. (b) OER energy profile on Co@N_4_-g at different densities calculated at 1.23 V *vs.* SHE. (c) pDOS of Co 3d orbitals at different densities. Reproduced with permission.^96^ Copyright 2025, American Chemical Society. HAADF-STEM images of Ir_1_/CoOOH (d) and Ir_1_/CoGaOOH (e). (f) Polarization curves of catalysts towards oxygen evolution in 1.0 M KOH electrolyte (top). Overpotentials of CoOOH, CoGaOOH, Iso-Ir_1_/CoOOH, and Nei-Ir_1_/CoGaOOH at a current density of 10 mA cm^−2^ (down). Reproduced with permission.^90^ Copyright 2024, Wiley VCH. (g) HAADF-STEM images of Ir_1_/Cu_0.3_Co_2.7_O_4_ with different Ir–Ir distances. (h) Polarization curves of catalysts at different scan cycles in 0.1 M HClO_4_ electrolyte. The displayed polarization curves are of the 1st, 200th, 400th, 600th, 800th, and 1000th cycle, respectively. Reproduced with permission.^91^ Copyright 2024, Springer Nature.

Increasing the density of single atoms will break the electronic symmetry of the support, and in this case the traditional d-band center theory is no longer applicable. Harrath *et al.*^96^ systematically investigated the effect of SAC density on OER overpotentials. For Co, Ni, and Cu SACs, the higher density lowered the overpotential by reducing the energy barrier for oxygen intermediate formation (Fig. 9a and b). In contrast, Fe SACs showed increased overpotential due to the rate-limiting OOH to O_2_ step. This divergence originates from support electronic restructuring under high SAC loading. DFT calculations revealed an anomalous d-band center behavior: although the d-band center shifted downward relative to the Fermi level, the OER activity improved. Charge density difference, spin density, and electron localization function analyses indicated that high-density SACs disrupt the symmetry of N-doped graphene, impede π-electron delocalization, and significantly raise the Fermi level, facilitating electron transfer from the support to metal sites (Fig. 9c). This accumulation of electrons on metal and adjacent N/C atoms suggests limitations of the conventional d-band theory in describing extreme electronic environments in SACs, underscoring the importance of spin state modulation.

Notably, spin states of Fe/Co SACs evolved with increasing density: the spin density of Co increased from 0*µ*_B_ in 1Co@N_4_-g to 1.03*µ*_B_ in 4Co@N_4_-g, indicating the LS to IS transition *via* long-range interactions, which optimized intermediate adsorption. At high densities, the OER mechanism for Co/Ni/Cu SACs shifted from the AEM to an intramolecular oxygen coupling (IMOC) pathway, *i.e.*, the OPM. This allows direct coupling of O intermediates on adjacent sites, significantly lowering the energy barrier for O_2_ generation.

Numerous studies have shown that the regulation of atomic density (atomic spacing) will significantly enhance the catalytic activity. Cao *et al.*^97^ synthesized high-density Ir single atoms (32 atoms per nm^2^) anchored on Co(OH)_2_*via* one-step electrodeposition. EXAFS indicated an initial Ir–Cl_6_ coordination environment. Co K-edge XANES showed a positive shift in the absorption edge after the OER, indicating increased oxidation of Co species, while Raman spectroscopy confirmed the oxidation of Co(OH)_2_ to CoOOH. The Ir active center transformed into an Ir–O_6_ configuration. Only high-density single atoms (≥32 atoms per nm^2^) can induce this support reconstruction, exposing Ir–O_6_ sites. This high-density induced Ir–O_6_ structure enhanced bonding orbital occupancy. COHP analysis showed a stronger Ir–O bond (ICOHP = −3.24 eV) compared to Co–O (−2.58 eV), confirming higher bonding orbital occupancy under high density, which reduced the energy barrier for the rate-determining step O* to OOH* and improved the OER activity, requiring only 173 mV overpotential to reach 10 mA cm^−2^. Ma *et al.*^90^ introduced Ga into CoOOH to increase the oxygen vacancy concentration and loaded high-density Ir SACs (81.5 ± 4.6 per 10 nm^2^) on CoGaOOH (Fig. 9d and e). Neighboring Ir atoms exhibited synergistic interactions superior to isolated Ir atoms. Nei-Ir_1_/CoGaOOH achieved a low overpotential of 170 mV at 10 mA cm^−2^ in pH = 11 electrolyte and remained stable for over 2000 hours (Fig. 9f). The mass activity reached 6.6 A mg_Ir_^−1^ at 300 mV overpotential, 1.43 times higher than that of Iso-Ir_1_/CoOOH (4.6 A mg_Ir_^−1^). Reaction pathway studies indicated that the proximal synergy between high-density Ir atoms stabilizes the OOH* intermediate *via* additional hydrogen bonding, significantly lowering the reaction barrier.

The interatomic distance also influences reaction selectivity. Pt dimers (2Pt) with a Pt–Pt distance of 2.6 Å carry a positive charge due to strong MSI, favoring H* adsorption and enhancing hydrogen evolution reaction (HER) activity. In contrast, Pt pairs (Pt_2_) with a shorter distance of 0.9 Å are more negatively charged and favor OOH* desorption, making them more selective toward the OER.^98^

The regulation of atomic density can also enhance the stability of the catalyst. High-density SACs can alter the local charge distribution and enhance the MSI to anchor the SACs. The highly active surface can reduce the concentration of OH^−^ in the microenvironment, preventing the dissolution and inactivation of SACs and supports. Zhang *et al.*^91^ synthesized Ir SACs with different Ir–Ir distances (Fig. 9g) and observed that Ir heteroatom-induced stabilization strongly depends on the distance between adjacent Ir single atoms. Shorter Ir–Ir distances enhanced the OER stability of cobalt oxides, with optimal stabilization at 0.6 nm. The catalyst retained stability at pH 1, with only a ∼20 mV increase in potential after 60 hours at 10 mA cm^−2^ (Fig. 9h). Theoretical calculations showed that shorter Ir–Ir distances increase migration energy, stabilizing surface Co atoms and effectively protecting the entire spinel oxide under acidic conditions. Cao *et al.*^92^ found that introducing highly active Ir single atoms suppressed Fe dissolution from the support, improving stability. The system remained stable for over 1000 hours at 200 mA cm^−2^ in pH 14 electrolyte, a phenomenon also observed in other Fe-based LDH systems. DFT indicated that Ir sites reduce the activity and enhance the stability of nearby Fe sites, while finite element simulations suggested that OH^−^ depletion around Ir sites due to rapid consumption also stabilizes distant Fe sites.

Quan *et al.*^99^ proposed an atomic-scale self-rearrangement strategy, converting unstable Ir precursors during electrolysis into stable high-density Ir single atoms (52 per 10 nm^2^) embedded in symmetry-broken CoCeOOH ultrathin nanosheets, forming CoCe–O–Ir_SA_. This material exhibited excellent OER activity, requiring only 187 mV to drive 100 mA cm^−2^, and operated stably in seawater electrolysis for 150 hours. Breaking support symmetry is key to achieving strong MSI and synthesizing high-density SACs, as it promotes electron accumulation on coordination atoms or generates oxygen vacancies for anchoring single atoms.

In summary, increasing the density of SACs not only raises the number of active sites per unit area but also significantly alters the electronic properties of metal centers and the adsorption/activation behavior of reaction intermediates through MSI and synergistic effects between neighboring metal sites. This can shift the reaction mechanism, thereby breaking the linear scaling relationships inherent in conventional AEM and providing an effective strategy for substantially improving the catalytic performance.

### 5.4 Spatial positioning

**Fig. 10:**
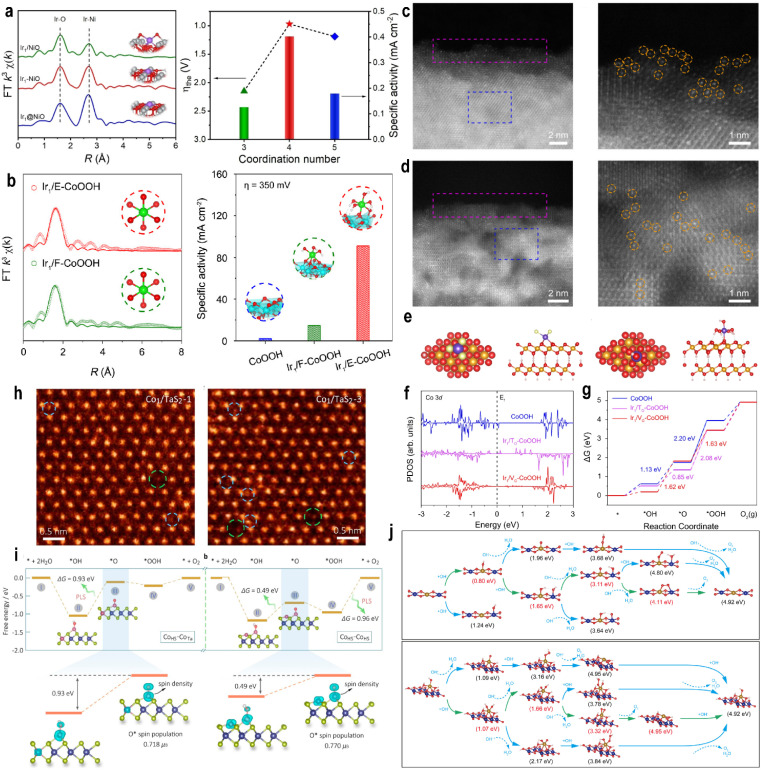
(a) Abstract diagram for optimizing the adsorption of intermediates by manipulating the second coordination shell of Ir single atoms for efficient water oxidation. Reproduced with permission.^100^ Copyright 2024, Wiley VCH. (b) Abstract diagram for modulating spatial distributions of single atoms on supports for enhanced oxygen evolution. (c) HAADF-STEM image of Ir_1_/E-CoOOH (left). (right) Localized magnified HAADF-STEM image of the pink rectangle in the left image. (d) HAADF-STEM image of Ir_1_/F-CoOOH (left). (right) Localized magnified HAADF-STEM image of the blue rectangle in the left image. Reproduced with permission.^102^ Copyright 2025, American Chemical Society. (e) Schematic structure model of Ir_1_/T_O_-CoOOH from top (left) and side (right) views. (f) Co 3d PDOS in CoOOH, Ir_1_/T_O_-CoOOH, and Ir_1_/V_O_-CoOOH. (g) Free energy diagrams. Reproduced with permission.^103^ Copyright 2022, Springer Nature. (h) HAADF-STEM images of the basal plane of a Co_1_/TaS_2_-1 monolayer and a Co_1_/TaS_2_-3 monolayer, respectively. (i) Free energy diagrams of the OER on the Co_HS_ site with neighboring Co_Ta_ and Co_HS_ atoms. The marked and enlarged parts: the pivotal step in the transformation from *OH to *O on the Co_HS_–Co_Ta_ and Co_HS_–Co_HS_ sites along with spin density distributions. Reproduced with permission.^106^ Copyright 2021, American Chemical Society. (j) Scheme of the OER mechanism and Gibbs free energy. (top) Planar-Fe–Co DSC and (down) stereo-Fe–Co DSC (blue: Co; yellow: Fe; red: O; white: H). Reproduced with permission.^107^ Copyright 2024, National Academy of Sciences.

Recent investigations into the spatial positioning of single atoms have revealed that the observed differences in catalytic activity primarily originate from variations in their local coordination structures. The specific atomic position directly determines the coordination environment, which in turn governs the MSI. These interactions critically influence the energy levels and electronic configurations of metal atom orbitals, thereby affecting their ability to achieve optimal matching and bonding with molecular orbitals of specific reactants. Wei *et al.*^100^ systematically investigated Ir SACs with varying coordination numbers on NiO supports, synthesizing three distinct structures identified through EXAFS and X-ray Photoelectron Spectroscopy (XPS) analyses: Ir_1_/NiO(3) (3-coordinate), Ir_1_-NiO(4) (4-coordinate), and Ir_1_@NiO(5) (5-coordinate). Among these, the 4-coordinate Ir_1_-NiO exhibited optimal OER activity with only 225 mV overpotential at 10 mA cm^−2^ (Fig. 10a). With increasing Ir–O–Ni coordination number, the Ni oxidation state is elevated, while the Ir oxidation state is decreased, indicating enhanced electron transfer from Ni to Ir. Computational analysis revealed higher d_*z*^2^_ orbital occupation in Ir_1_@NiO (1.01 electrons) compared to Ir_1_/NiO (0.95 electrons), consistent with the e_g_ orbital filling descriptor principle where values closer to 1 correspond to peak OER activity. The increased d_*z*^2^_ orbital population in optimal configurations facilitates antibonding orbital filling in OOH* intermediates, thereby weakening the adsorption strength and improving the OER performance.

In complementary work, Kumar *et al.*^101^ reported that partially embedded Ir atoms (Ir_emb_-NiO) exhibited superior OER performance (*η*_100_ = 320 ± 10 mV overpotential) compared to surface-adsorbed Ir atoms (Ir_ads_-NiO, *η*_100_ = 342 ± 8 mV) in alkaline media. *Operando* X-ray absorption spectroscopy attributed this enhancement to stronger MSI in Ir_emb_-NiO, which promoted the formation of active NiOOH species. The embedded configuration showed doubled Ir–Ni second-shell coordination compared to surface-adsorbed ones, with the strong MSI effectively stabilizing Ir atoms against dissolution during 20 hour operation at 20 mA cm^−2^.

The spatial position determines the coordination saturation and exposure degree of the metal centers. This section will discuss representative studies on controlling single-atom positioning and present synthetic methodologies for SACs.

In CoOOH, there are oxygen vacancies and triple-hollow sites, which facilitate the study of the sources of the activity differences of single atoms at different positions. In a representative work by Liu *et al.*,^102^ the coordination numbers of Ir–O for both Ir_1_/F-CoOOH and Ir_1_/E-CoOOH were fitted to be about 6.0, suggesting that the Ir centers in both samples were coordinated with six O atoms, forming an IrO_6_ octahedral structure (Fig. 10b). Ir single atoms were anchored at distinct sites on CoOOH supports: boundary positions (Ir_1_/E-CoOOH) and surface positions (Ir_1_/F-CoOOH) (Fig. 10c and d). The superior OER activity of Ir_1_/E-CoOOH was attributed to hydrogen bonding interactions that optimize adsorption energetics. The anchoring mechanism was explained through electrostatic interactions during electrodeposition: under anodic conditions, Ir(OH)_6_^*x*−^ species are attracted to positively charged oxygen vacancies, while under cathodic conditions, IrCl^*x*+^ cations are drawn to negatively charged triple-hollow sites, forming IrCl_3_O_3_ configurations that subsequently transform into IrO_*x*_ coordination structures under oxidizing potentials. This fundamental understanding has enabled precise site-selective anchoring of single atoms. Based on the above theories, a series of methods for anchoring Ir on CoOOH have been reported. Zhang *et al.*^103^ employed targeted electrochemical deposition to create Ir_1_/T_O_-CoOOH (triple-hollow sites) and Ir_1_/V_O_-CoOOH (oxygen vacancy sites). While both configurations enhanced OER performance compared to pure CoOOH (340 mV at 10 mA cm^−2^), Ir_1_/V_O_-CoOOH exhibited the most significant improvement (200 mV) (Fig. 10e). In Ir_1_/T_O_-CoOOH, strong Ir–Co charge interaction reduced the Co 3d band gap, increasing electrophilicity for intermediate adsorption. In Ir_1_/V_O_-CoOOH, the charge effect between Ir and Co is relatively weak (Fig. 10f), and the Ir-coordinated hydroxyl group (Ir-OH) formed hydrogen bonds with OER intermediates, stabilizing transition states through conformational effects (Fig. 10g).

Feng *et al.*^104^ further compared lattice-incorporated (Ir_1_/CoOOH_lat_) and surface-adsorbed (Ir_1_/CoOOH_sur_) Ir SACs. XANES and EXAFS analyses revealed significant Ir to Co electron transfer in the lattice-incorporated system, resulting in higher Ir oxidation states. Despite both systems primarily utilizing Co sites for the OER, the Ir_1_/CoOOH_sur_ configuration demonstrated superior activity, with Ir atoms stabilizing OOH* species through hydrogen bonding.

Different metal atoms can also be loaded on the three-dimensional vacancies and oxygen vacancies. For example, Ma *et al.*^104^ developed a Ru_T_Ir_V_/CoOOH catalyst through a wet-chemical method that selectively anchors Ru single atoms at triple hollow sites, combined with electrodeposition of Ir atoms at oxygen vacancy sites. Compared to Ir_T_Ru_V_/CoOOH, the Ru_T_Ir_V_/CoOOH configuration demonstrated superior OER performance. The d-band center of CoOOH was measured at −1.24 eV, which shifted to −1.88 eV in Ru_T_/CoOOH after Ru loading, while the introduction of Ir in Ru_T_Ir_V_/CoOOH caused negligible further shift. This electronic behavior confirms the stronger MSI between Ru and CoOOH than between Ir and CoOOH. Specifically, Ru atoms at triple hollow sites interact with three oxygen atoms from the CoOOH lattice, whereas Ir atoms at oxygen vacancy sites coordinate with only one oxygen atom. The increased number of bridging oxygen atoms enhances the MSI for Ru compared to Ir. *In situ* spectroscopic characterization and mechanistic studies revealed that Ru atoms serve as the primary active centers for adsorbing key reaction intermediates, while Ir atoms at oxygen vacancy sites stabilize OOH* species through hydrogen bonding interactions, thereby facilitating the oxygen evolution process. The aforementioned studies consistently demonstrate that Ir single atoms anchored at oxygen vacancy sites, despite exhibiting relatively weaker MSI, display superior catalytic activity for the OER. This enhanced performance is attributed to their ability to stabilize intermediates through hydrogen bonding between their coordinated OH ligands and adsorbed species on adjacent Co sites. This stabilization mechanism effectively optimizes the adsorption energetics of reaction intermediates, thereby significantly improving the overall OER activity of the CoOOH support.

In their investigation of Ir single atoms on CoOOH, Yang *et al.*^105^ similarly observed higher OER activity for Ir_1_/CoOOH_sur_ (453 mV@10 mA cm^−2^) compared to Ir_1_/CoOOH_lat_ (461 mV). However, their synthesis employed a distinct low-temperature photochemical strategy: CoOOH was dispersed in an IrCl_3_ aqueous solution under ultrasonication, rapidly frozen in a liquid nitrogen bath, and subsequently irradiated with UV light at cryogenic temperatures. Notably, the active site in Ir_1_/CoOOH_sur_ was identified as a surface [IrO_5_] configuration, contrasting with the Co sites in CoOOH and Ir_1_/CoOOH_lat_. This structural difference originates from enhanced electron transfer between the [IrO_5_] and adjacent oxygen atoms, resulting in a higher positive charge on Ir atoms compared to the [IrO_6_] octahedra in Ir_1_/CoOOH_lat_. This observation appears contradictory to previous reports where catalysts with weaker MSI generally exhibited superior OER activity. Such seemingly conflicting conclusions highlight the intricate complexity underlying the spatial control of single-atom positioning in catalytic systems. When Ir is the main active center, the moderate MSI optimizes the d-orbital electron occupancy of Ir, thereby balancing the adsorption and desorption of reaction intermediates to achieve the highest intrinsic activity; when Ir acts as a promoter, the weaker MSI can retain Ir in a specific electronic state, thereby maximizing hydrogen bonding stabilization and promoting Co as the primary active center.

In dual-atom systems, spatial arrangement creates unique synergistic effects. Li *et al.*^106^ synthesized ferromagnetic single Co atom catalysts on TaS_2_ monolayers (Co_1_/TaS_2_). A single Co atom adsorbed at the hollow site (Co_HS_) with spin-polarized electronic states serves as the active site for the OER, whose spin density can be regulated by its neighboring single Co site at Ta substitution sites (Co_Ta_) *via* tuning the Co loading. The Co loadings of Co_1_/TaS_2_-1 and Co_1_/TaS_2_-3 were measured to be 2.2% and 5.7%, respectively (Fig. 10h). An optimized spin density of Co_HS_ results in optimized O* binding energy (Fig. 10i). Similarly, Zhang *et al.*^107^ synthesized two distinct Fe single-atom configurations on a CoOOH support: a planar Fe–Co dual-site catalyst (planar-Fe–Co DSC) and a stereoscopic Fe–Co structure (stereo-Fe–Co DSC). Electrochemical measurements revealed that the planar configuration achieved a lower overpotential of 190 mV at 10 mA cm^−2^, compared to 210 mV for the stereoscopic analogue. The PDOS analysis demonstrated that in the planar-Fe–Co DSC, the d_*z*^2^_ orbital of the Fe atom and the d_*xz*_/d_*yz*_ orbitals of the adjacent Co atom synergistically contribute to the HOMO. This orbital synergy facilitates the steps of oxygen molecule dissociation. In contrast, the stereoscopic configuration exhibits dominant involvement of the d_*xz*_/d_*yz*_ orbitals in the evolution of reaction intermediates, resulting in a primary active center localized at the Fe atom with limited cooperative effects.

These findings are well explained by frontier molecular orbital theory. As shown in Fig. 10j, in the planar Fe–Co DSC, both Fe and Co atoms expose accessible orbitals for adsorbate interaction, leading to a dual-atom OPM. Conversely, the stereo-Fe–Co DSC relies solely on Fe as the active center, where the d_*xz*_/d_*yz*_ orbitals independently adsorb two oxygenated intermediates, following a single-atom OPM.

Collectively, these studies establish that the spatial position of single atoms precisely controls their coordination environment, which subsequently tunes the MSI. These interactions optimize adsorption behavior through electronic modulation, create synergistic effects that enhance intermediate stabilization, and in some cases trigger novel reaction pathways, ultimately leading to improved OER activity and stability.

### 5.5 Defect engineering

Defect engineering plays a crucial role in adjusting MSI in SACs. The strategic introduction of vacancies and defects enables precise control over the local coordination environment in both the first and second coordination shells of metal active centers. This microenvironment engineering significantly influences electron transfer and spatial interactions between metal centers and supports, consequently altering the energy levels and distribution of frontier orbitals.

These electronic modifications directly affect the interaction between SACs and adsorbates, ultimately optimizing adsorption energies and enhancing catalytic activity. Liu *et al.*^32^ investigated the stability of Au nanoclusters on CeO_2_ supports, demonstrating that clusters anchored at step edges exhibited superior stability compared to those on the planar surface, which tended to aggregate.

This stabilization mechanism originates from the dynamic formation of oxygen vacancies at step edges, which create new adsorption sites and deep potential wells for Au nanoparticles. Studies have shown that defect engineering can enhance the loading stability of SACs and alter the adsorption configuration by changing the coordination structure of the first shell layer and breaking the charge symmetry of supports. Wang *et al.*^108^ anchored Pt single atoms at *in situ* generated iron cation vacancies in layered α-Ni_2/3_Fe_1/3_(OH)_2_ through H_2_PtCl_6_ oxidation of Fe^2+^. Mechanistic studies revealed that Pt atoms at iron cation vacancies activate adjacent Ni atoms as OER active sites, optimizing intermediate adsorption energies and enhancing OER performance.

**Fig. 11:**
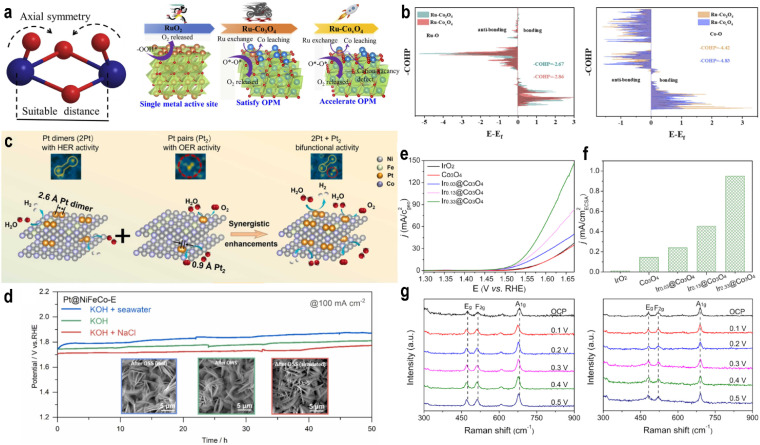
(a) O–O coupling promoted by dual active sites and reaction pathway schematics for RuO_2_, Ru-Co_3_O_4_, and Ru-Co_v_O_4_. Ru-Co_3_O_4_ contains only Ru single atoms, while Ru-Co_v_O_4_ incorporates both Ru single-atom and cationic defects. (b) COHP of (left) Ru–O and (right) Co–O bonds. Reproduced with permission.^109^ Copyright 2025, Elsevier. (c) Schematic illustration of Pt dimers and Pt pairs on NiFeCo-E and their bifunctionality towards water splitting catalysis. (d) Chronopotentiometric curves of the overall splitting of alkalized freshwater, simulated seawater, and real seawater for Pt@NiFeCo-E//Pt@NiFeCo-E at 100 mA cm^−2^ for 50 h; the insets show the morphologies after tests. Reproduced with permission.^98^ Copyright 2023, Elsevier. (e) LSV curves of Ir_*x*_@Co_3_O_4_, Co_3_O_4_, and IrO_2_ in 1 M KOH. (f) ECSA-normalized current density of Ir_*x*_@Co_3_O_4_, Co_3_O_4_, and IrO_2_ at the potential of 1.65 V *vs.* RHE. (g) *In situ* Raman spectra for the OER occurring on (left) Co_3_O_4_ and (right) Ir_0.33_@Co_3_O_4_ at different overpotentials. Reproduced with permission.^111^ Copyright 2025, American Chemical Society.

Advancing beyond simple vacancy creation, Zhang *et al.*^109^ created tetrahedral Co cation defects while selectively anchoring Ru SACs at octahedral cobalt sites in Co_3_O_4_(Ru-Co_v_O_4_). The cation defects act as electron acceptors, promoting electron transfer from Ru to Co and enhancing density of states overlap. This electronic interaction strengthens orbital hybridization and MSI. As shown in Fig. 11b, the increased absolute ICOHP values for Co–O bonds (from 3.65 in Co_3_O_4_ to 4.42 in Ru-Co_3_O_4_) indicate enhanced bond formation through electron filling of bonding orbitals. The optimized electronic structure reduces the energy barrier for O* intermediate coupling in the OPM (Fig. 11a), achieving an exceptionally low overpotential of 150 mV at 10 mA cm^−2^, significantly outperforming both Co_3_O_4_ (510 mV) and conventional Ru-Co_3_O_4_ (200 mV), while retaining outstanding stability with only 0.35 mV h^−1^ degradation at 10 mA cm^−2^.

Zhang *et al.*^110^ proposed a novel catalytic center theory where specific coordination configurations formed by single-atom metal species and carbon defects exhibit distinct electronic states and electrochemical activities for different reactions. Nickel atoms trapped in graphene defects show variable coordination environments: aNi@D5775 with seven carbon coordinations demonstrates excellent HER activity, while aNi@Di-vacancy with four carbon coordinations exhibits superior OER performance. Similarly, Pt diatomic active sites on cation-vacancy-rich NiFeCo-LDH show configuration-dependent selectivity, with Pt dimers favoring the HER and Pt pairs preferring the OER (Fig. 11c). As shown in Fig. 11d, electrolytic cells composed of Pt SACs with different spacings can electrolyze seawater for 50 hours with stability.^98^

Chen *et al.*^111^ constructed Ir SACs on Co_3_O_4_ substrates *via* thermal decomposition as Ir_0.33_@Co_3_O_4_. Ir substitution at Co^3+^ sites weakens Co–O bonds and promotes oxygen vacancy formation, creating unique electronic and geometric structures. DFT calculations revealed that the synergistic effect between oxygen vacancies and Ir single atoms shifts both metal d-band and O 2p-band centers toward the Fermi level, inducing electron transfer from Co active sites to adjacent Ir single atoms. The MSI between Ir SACs and Co_3_O_4_ not only suppresses Co_3_O_4_ structural transformation but also stabilizes Ir single atoms, achieving an overpotential of 296 mV at 10 mA cm^−2^ with significantly enhanced stability compared to pure Co_3_O_4_ (Fig. 11e and f). As shown in Fig. 11g, *in situ* Raman spectroscopy demonstrated that while [CoO_6_] octahedra in Co_3_O_4_ undergo significant structural transformation during the OER (evidenced by negative shifts of A_1g_ modes at 0.1–0.5 V overpotentials), Ir_0.33_@Co_3_O_4_ retained stable peak positions and intensities.

Synergistic MSIs between SACs and nanoparticles have also been reported. For instance, TiO_2_ nanowires decorated with both Ir nanoparticles and Sr single atoms require only 250 mV and 32 mV overpotentials to reach 10 mA cm^−2^ for the OER and HER in acidic media, respectively.^112^ DFT calculations indicate that oxygen vacancies and Sr adsorption promote charge transfer from TiO_2_ to Ir nanoparticles, optimizing the adsorption of OER and HER intermediates while enhancing Ir nanoparticle stability through MSI.

In summary, defect engineering enables precise manipulation of SAC performance through controlled modification of coordination environments. By engineering vacancies and defects, researchers can effectively tune electron transfer behavior and spatial interactions between metal centers and supports, thereby regulating frontier orbital characteristics to optimize adsorbate interactions and ultimately enhance catalytic activity.

## 6. Conclusion and perspectives

In this review, we have systematically evaluated the development of descriptors for the MSI. We have examined descriptors for assessing the stability of SACs, revealing that optimal stability is achieved at intermediate MSI strength, following a Sabatier-type principle. The strength of these interactions can be significantly modulated by reaction potentials. Regarding adsorption capability descriptors, we have discussed the limitations of universal parameters such as the d-band center and Bader charge. While valuable within specific systems, these parameters often fail to accurately describe the electronic orbitals of SACs that exhibit free-atom-like characteristics. Alternatively, descriptors derived from molecular orbital theory, particularly the occupancy and energy of specific orbitals (*e.g.*, *z*-axis orbitals governing axial adsorption or e_g_ orbitals showing volcano-type relationships with OER activity) that demonstrate enhanced predictive capability. Furthermore, we have highlighted the significant contribution of electrostatic adsorption for oxygen species containing lone-pair electrons during the OER. Consequently, effective descriptors inherently form composite models that integrate the two fundamental components of chemical bonding: covalent interactions (comprising orbital overlap, energy matching, and symmetry alignment) and electrostatic interactions (coulombic forces).

Regardless of whether it is through spin configuration, axial coordination, or defect engineering, the essence is to precisely control the crystal field through supports, thereby altering the energy level splitting, energy levels, and electron occupation of the metal d orbitals. The goal of this orbital engineering is to fine-tune the adsorption energies of OER intermediates (OH, O, and OOH*) by tailoring the MSI.

This enables breaking of the linear scaling relationships that constrain catalytic activity in the AEM, directing reactions along alternative pathways like the LOM or OPM that exhibit lower overpotentials. These insights provide a robust foundation for the rational design of high-performance SACs.

**Fig. 12:**
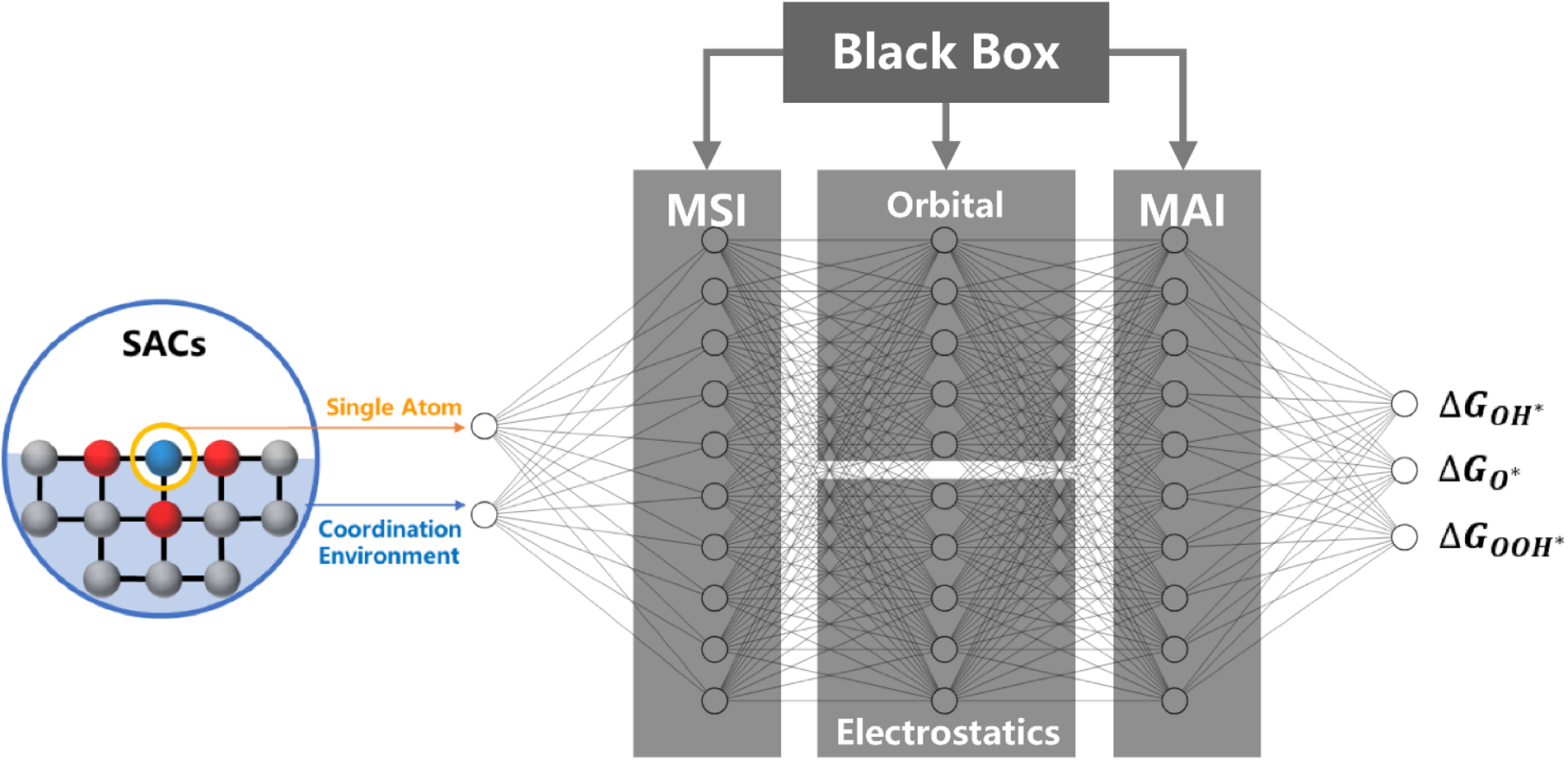
Schematic for the black box from coordination shell regulation to adsorption energy in SAC design.

The intermediate adsorption energy of the catalyst directly affects the reaction activity. *In situ* and *ex situ* characterization studies help us understand the real structure of the catalyst. However, how the support environment of SACs specifically influences the intermediate adsorption energy remains a black box in the design theory of SACs. The support regulates the microscopic coordination environment of single atoms, and the change in the coordination environment affects the energy level and shape of the frontier molecular orbitals (FMOs) of SACs. The alteration of FMOs determines the strength of the adsorption energy. The influence of the electronic orbitals of single atoms regulated by the MSI on the adsorption energy provides an analytical approach for the black box. However, there are still problems such as the unclear microscopic mechanism of the interaction between single atoms and the support surface and the incomplete regulation and characterization of the microscopic environment of single atoms. Therefore, we need more advanced synthesis methods, characterization techniques, and theoretical simulation technologies to quantitatively analyze how the MSI affects the electronic orbital state of single atoms and thereby optimize the adsorption energy of SACs (Fig. 12).

### 6.1 Advanced synthesis method

While a variety of synthetic methods have been established, such as the pyrolysis of metal–organic frameworks (MOFs), “host–guest” strategies, “top-down” strategies, zeolite confinement, *etc.*,^113^ the next frontier is achieving atomic-level precision. To truly test and apply the principles discussed in this review, we need to develop efficient and precise synthesis methods that can deliberately control the local coordination environment. These methods should allow for the precise synthesis of SACs with targeted spin configurations, specific axial ligands, tunable interatomic distances, or selective anchoring at desired defect sites. For example, the cubic Co(CN)_3_ (Co(CN)_3_-Cub) with five-coordinated Co sites exhibit an outstanding ORR half-wave potential (*E*_1/2_) of 0.90 V *vs.* RHE, considerably exceeding the values of three-coordinated octahedral Co(CN)_3_ (Co(CN)_3_-Oct). For Co(CN)_3_-Cub, its d-band center principle Fermi level is conducive to OH* desorption, showing higher activity.^114^ By combining high spatial–temporal resolution characterization and simulation, and answering these problems, we can gradually understand the influence of MSI on SACs and thereby fill the cognitive black box from the real structure to the adsorption energy.

### 6.2 Precise characterization and simulation

To directly validate and refine the “structure–adsorption” framework proposed in this review, it is essential to develop *in situ* characterization techniques with high spatiotemporal resolution. The goal is not only to achieve precise detection of atomic structures but also, more importantly, to visualize the dynamic evolution of the electronic states that govern adsorption.^115^ Specifically, quantitative electron microscopy imaging of single atoms and *in situ* observations of their dynamic behavior on supports are required to fundamentally understand the MSI under realistic reaction conditions. During catalytic processes, the support often undergoes dynamic restructuring, which subsequently alters the local coordination environment and orbital states of the single atoms. Advanced characterization enables real-time, atomic-level tracking of key parameters such as coordination number, oxidation state, and bond length during the reaction. For instance, SACs may undergo dynamic anchoring or form clusters under OER conditions.^12^ Through interpretation of *in situ* spectroscopic data, the dynamic coordination changes in single-atom sites and the evolution of MSI can be elucidated, contributing to a more definitive structure–activity relationship.

Furthermore, a deeper understanding of molecular orbital coupling in SACs is needed. UV-vis and Mott–Schottky analyses provide band-edge information on semiconductor supports; frontier orbital positions of molecules/complexes should be obtained *via* UPS or DFT. Molecular adsorption experiments can further assist in analyzing the frontier orbitals and their charge distribution. Beyond orbital energetics, electron spin states play a critical role in catalysis. While *in situ*^57^Fe Mössbauer spectroscopy can analyze the spin state of Fe-based SACs under working conditions, analogous techniques for probing local spin states in other metal single-atom systems under working conditions are currently lacking and represent an important direction for future research.

In addition to advanced experimental techniques, theoretical simulations that more closely reflect realistic reaction conditions are necessary. Unlike thermocatalysis, electrochemical reactions involve charge accumulation at electrode surfaces, leading to differences between *in situ* and *ex situ* electronic structures. Calculating the electronic states (frontier orbitals, orbital energy levels, orbital occupancy, *etc*.) of SACs under different reaction potentials is very helpful for understanding the influence of orbital overlap and bonding on adsorption energy during the reaction process. Moreover, high time-resolution simulations can provide detailed insights into the dynamic processes of MSI.

### 6.3 Data-assisted design

MSI has a direct and quantifiable influence on the electronic structure of SACs. Through precise modulation of key coordination parameters, including the electronegativity of coordinating atoms, bond lengths, and spatial configuration, MSI effectively tunes the energy levels, occupancy, and symmetry of the metal center's d orbitals. These electronic modifications subsequently alter the adsorption strength of reaction intermediates on the active site, further determining the catalytic activity and selectivity.

This well-defined tunability provides an ideal source of accurately labeled data for constructing high-quality databases. Such a database enables machine learning methods to establish robust quantitative structure–activity relationships based on chemical meaningful descriptors. A comprehensive SAC research not only deepens the fundamental understanding of MSI but also, combined with precise synthetic methodologies, facilitates the rational design of catalysts with optimized adsorption energies, significantly accelerating catalyst development. Furthermore, the database can be utilized to predict phase diagrams and Pourbaix diagrams, thereby identifying the most stable structures of SACs under synthesis or operational conditions. This capability provides critical support for interpreting experimental characterization results.

Currently, the research priority is to systematically accumulate *in situ* spectroscopic data and theoretical adsorption energies across diverse metal centers, coordination environments, and reaction conditions. These efforts aim to build reliable training sets for predictive models. The ultimate goal is to establish a universal design theory that integrates electronic, thermodynamic, and kinetic parameters for SACs.

This review consolidates key descriptors of MSI that govern the stability and activity of SACs. It establishes a structure-adsorption framework highlighting two primary adsorption modes in SACs: covalent bonding through orbital hybridization and electrostatic interactions. Subsequently, examples of how MSI can regulate the adsorption energy of single atomic sites are presented. This highlights the significant role of the microscopic coordination structure directly influenced by MSI in regulating the electronic structure of SACs. To gain a comprehensive understanding of MSI and design efficient OER catalysts with optimized active site structures, it is crucial to focus on how MSI influences frontier orbital energy/geometry and charge distribution. This requires advanced synthetic methodologies to obtain SACs with clear coordination structures and through precise characterization and simulation to understand the changes during the OER process.

## Author contributions

All of the authors contributed to the manuscript preparation. H. C. and J. Z. conceived the outline of the manuscript. H. C. wrote the original draft of the manuscript. J. Z. and C. C. discussed and helped revise the manuscript.

## Conflicts of interest

There are no conflicts to declare.

## Data Availability

No primary research results, software or code have been included and no new data were generated or analysed as part of this review.
